# High-resolution epitope mapping and characterization of SARS-CoV-2 antibodies in large cohorts of subjects with COVID-19

**DOI:** 10.1038/s42003-021-02835-2

**Published:** 2021-11-22

**Authors:** Winston A. Haynes, Kathy Kamath, Joel Bozekowski, Elisabeth Baum-Jones, Melissa Campbell, Arnau Casanovas-Massana, Patrick S. Daugherty, Charles S. Dela Cruz, Abhilash Dhal, Shelli F. Farhadian, Lynn Fitzgibbons, John Fournier, Michael Jhatro, Gregory Jordan, Jon Klein, Carolina Lucas, Debra Kessler, Larry L. Luchsinger, Brian Martinez, M. Catherine Muenker, Lauren Pischel, Jack Reifert, Jaymie R. Sawyer, Rebecca Waitz, Elsio A. Wunder, Minlu Zhang, Yale IMPACT Team, Kelly Anastasio, Michael H. Askenase, Natasha C. Balkcom, Maria Batsu, Santos Bermejo, Kristina Brower, Molly L. Bucklin, Staci Cahill, Yiyun Cao, Michael Chiorazzi, Caitlin J. Chun, Rupak Datta, Giuseppe DeIuliis, Coriann E. Dorgay, Rebecca Earnest, John Fournier, Bertie Geng, Ryan Handoko, William Khoury-Hanold, Roy Herbst, Lynda Knaggs, Maxine Kuang, Sarah Lapidus, Zitong Lin, Peiwen Lu, Tianyang Mao, Anjelica Martin, Irene Matos, David McDonald, Maksym Minasyan, Adam J. Moore, Nida Naushad, Allison Nelson, Jessica Nouws, Angela Nunez, Hong-Jai Park, Xiaohua Peng, Alexander James Robertson, Tyler Rice, Kadi-Ann Rose, Wade Schulz, Lorenzo Sewanan, Lokesh Sharma, Denise Shepard, Julio Silva, Michael Simonov, Mikhail Smolgovsky, Nicole Sonnert, Ariktha Srivathsan, Yvette Strong, Codruta Todeasa, Jordan Valdez, Sofia Velazquez, Pavithra Vijayakumar, Elizabeth B. White, Alice Zhao, Akiko Iwasaki, Albert Ko, John C. Shon

**Affiliations:** 1 Serimmune, Inc., Goleta, CA USA; 2 Department of Epidemiology of Microbial Diseases, Yale School of Public Health, New Haven, CT USA; 3 Department of Medicine, Section of Pulmonary and Critical Care Medicine, Yale University School of Medicine, New Haven, CT USA; 4 Department of Medicine, Section of Infectious Diseases, Yale University School of Medicine, New Haven, CT USA; 5 Santa Barbara Cottage Hospital, Santa Barbara, CA USA; 6 Department of Immunobiology, Yale University School of Medicine, New Haven, CT USA; 7 New York Blood Center, New York, NY USA; 8 Howard Hughes Medical Institute, Chevy Chase, MD USA; 9 Yale Center for Clinical Investigation, Yale University School of Medicine, New Haven, CT USA; 10 Department of Neurology, Yale University School of Medicine, New Haven, CT USA; 11 Yale University School of Medicine, New Haven, CT USA; 12 Department of Laboratory Medicine, Yale University School of Medicine, New Haven, CT USA; 13 Center for Outcomes Research and Evaluation, Yale-New Haven Hospital, New Haven, CT USA

**Keywords:** Adaptive immunity, Diagnostic markers, Viral infection, Viral infection, Data mining

## Abstract

As Severe Acute Respiratory Syndrome Coronavirus 2 (SARS-CoV-2) continues to spread, characterization of its antibody epitopes, emerging strains, related coronaviruses, and even the human proteome in naturally infected patients can guide the development of effective vaccines and therapies. Since traditional epitope identification tools are dependent upon pre-defined peptide sequences, they are not readily adaptable to diverse viral proteomes. The Serum Epitope Repertoire Analysis (SERA) platform leverages a high diversity random bacterial display library to identify proteome-independent epitope binding specificities which are then analyzed in the context of organisms of interest. When evaluating immune response in the context of SARS-CoV-2, we identify dominant epitope regions and motifs which demonstrate potential to classify mild from severe disease and relate to neutralization activity. We highlight SARS-CoV-2 epitopes that are cross-reactive with other coronaviruses and demonstrate decreased epitope signal for mutant SARS-CoV-2 strains. Collectively, the evolution of SARS-CoV-2 mutants towards reduced antibody response highlight the importance of data-driven development of the vaccines and therapies to treat COVID-19.

## Abstract (web summary)

Using a high throughput, random bacterial peptide display approach applied to patient serum samples, Haynes, Kamath, Bozekowski et al identify the antigens and epitopes that elicit a SARS-CoV-2 humoral response. They identify differences depending on disease severity and further in silico analysis suggests decreased epitope signal for Q677P but not for D614G mutant SARSCoV-2 strains.

## Introduction

The novel coronavirus SARS-CoV-2 global pandemic has affected millions of people world-wide and led to a major healthcare crisis. Considerable research has gone into understanding the myriad symptoms that are seen in patients as well as the stark contrast between the large number of mild or asymptomatic cases and the staggering death toll around the world^1–5^. Determining the factors that contribute to different disease manifestations, severity and immunity is critical to adequate therapeutic intervention, improved patient outcomes, and vaccine design.

One avenue that is being extensively explored is the degree to which an immune response to the virus protects, or harms, an individual. Although it is possible that a pre-existing exposure to common coronaviruses may have a protective role during SARS-CoV-2 infection^6,7^, it has also been proposed that antibodies to SARS-CoV-2 may sometimes be directly pathogenic or lead to the generation of auto-reactive antibodies^8–12^. With millions of cases extant, and based on current trends, millions more in the coming months, it is critical that patients be accurately assessed not just for infection but also for the potential of severe disease progression, allowing timely application of treatments for best outcomes. Of considerable concern as well is the specter of a combined SARS-CoV-2/influenza season with the need to rapidly differentiate between multiple viral infections^13,14^. In addition, a growing number of COVID-19 patients who had expected to fully recover have not, with symptoms that linger far past the expected recovery period and cause significant disruption to their lives as well as an extended need for healthcare. The number of “long-haulers” is not currently clear but the need to elucidate the role of a disrupted immune system in their illness is pressing^15–17^.

Along with the initial step of defining an effective vaccine for the immediate crisis, factors such as viral mutation rate and the uncertainty of long-term immunity could play a large role in ongoing management. It is unclear if it will be possible to develop “sterilizing immunity” to the virus, thus preventing infection completely^18–20^. A yearly “flu-type” immunization would necessitate continued surveillance of both viral evolution and patients’ yearly immune responses to keep transmission and mortality to a minimum^21^.

Many different groups have examined antibody responses to SARS-CoV-2, exploring correlation with disease severity, duration of humoral response, and the neutralizing capacity of response^3,22,23^. Most of these methods have been limited to quantitative assessment of humoral response to whole proteins or large domains of spike and nucleoprotein. Peptide and phage display libraries have also been used to capture higher resolution epitope patterns associated with disease but are limited to characterization of linear epitope signal and in their ability to make clinical seropositivity assessments^4,24,25^. We present in this paper the application of Serum Epitope Repertoire Analysis (SERA), a high throughput, random bacterial peptide display technology that enables assessment of SARS-CoV-2 seropositivity and high-resolution mapping of epitopes across any arbitrary proteome, including wild-type SARS-CoV-2, its mutant strains, common coronaviruses, and the human proteome.

We have leveraged over 1500 pre-pandemic immune repertoires and over 500 COVID-19 cases to identify the antigens and epitopes that elicit a SARS-CoV-2 humoral response. We show that while antibody profiles of individuals are heterogeneous, epitope-level resolution enables a range of analyses and visualizations, from the earliest epitopes to elicit an antibody response, to identification of epitopes that may be important for neutralization or immunity. Combining epitope motifs into a panel yields a diagnostic classifier that distinguished nucleic acid test (NAT) positive cases from controls with accuracy comparable to serological tests in current use. Differences in the quantity and quality of epitopes in mild versus moderate and severe disease can be seen at sites of biological and clinical interest. In silico analysis of epitope repertoires on wild-type and mutant SARS-CoV-2 proteins suggests that some mutations may result in loss of antibody reactivity to mutant SARS-CoV-2 infections while analysis against the human proteome identified SARS-CoV-2 antibodies that may cross-react with human proteins and contribute to disease pathogenesis. These capabilities are all possible through informatics analysis of a single assay that requires a minimal amount of serum from each subject.

## Results

### SERA screening of COVID-19 serum

We applied SERA to discover and validate SARS-CoV-2 antigens and epitopes across the complete viral proteome from 779 COVID-19 serum samples taken from 579 unique subjects while additionally leveraging a large database of pre-pandemic controls (Table 1). The majority of the subjects were confirmed SARS-CoV-2 positive by NAT. For Cohorts I, II, and III, extensive characterization was available for covariates that included disease severity, date of symptom onset, and in many cases, serological testing (Supplementary Data 1).

**Table 1:** SARS-CoV-2 cohorts used for epitope motif discovery.

SARS-CoV-2 cohorts			Training set		Test set		COVID-19 test
			# of donors	# of samples	# of donors	# of samples	
Cohort I	Yale	Inpatient	91	153	98	177	NAT and/or serology
		Healthcare Workers	4	4	6	9	NAT and/or serology
		Outpatient	4	4	5	5	Serology or symptoms
Cohort II	LabCorp	Inpatient			188	235	NAT
		Outpatient			10	10	Serology
Cohort III	SBCH	In and outpatient			73	82	NAT, Serology
Cohort IV	BioIVT	In and outpatient			21	21	Serology (20), NAT (1)
Cohort V	BCA	Asymptomatic/mild			79	79	Serology/SERA
	Total		99	161	480	618	
	Pre-pandemic Controls	IgG	497	497	1500	1500	
		IgM	430	430	1498	1498	

**Fig. 1:**
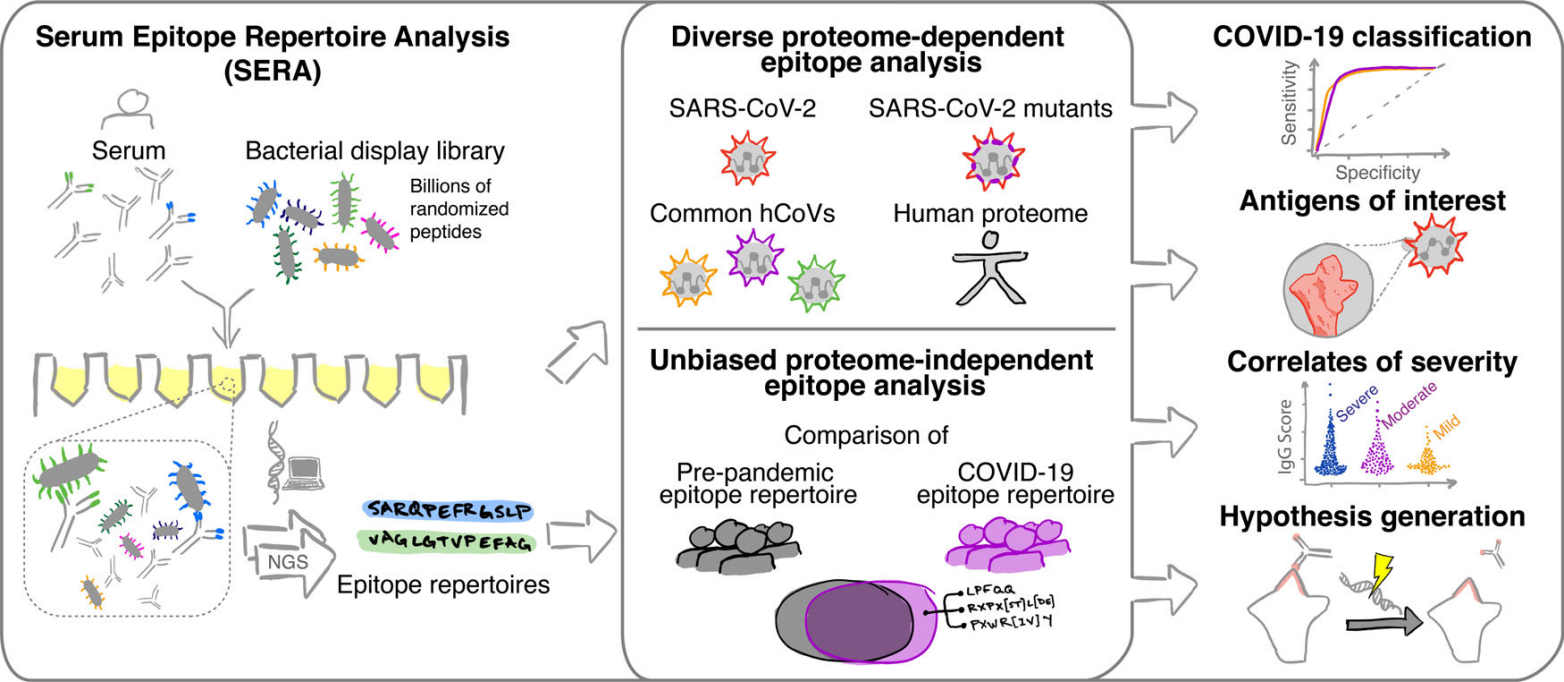
The Serum Epitope Repertoire Analysis (SERA) platform enables high-resolution mapping of SARS-CoV-2 antibody repertoires. The SERA assay results in a set of ~1 million unique peptides, the “epitope repertoire”, for each individual. Repertoires were deposited in a database and compared with pre-pandemic controls to identify conserved epitopes in SARS-CoV-2 using proteome-dependent and -independent bioinformatic methods. SERA enables analysis of COVID-19 repertoires against any proteome including mutant SARS-CoV-2 strains, human common coronaviruses and the human proteome for discovery of potential autoantigens. The identified epitope signatures can be used to build diagnostic classifiers, to identify correlates of disease severity, and to develop hypotheses based on cases with specific symptoms and/or disease course (neurological, GI, cardio e.g.).

Patient samples were all screened using the published SERA assay, which enables high throughput characterization of antibody epitopes (Fig. 1)^26,27^. In brief, serum or plasma is incubated with the randomized bacterial display peptide library; antibodies bind to peptides that mimic their natural epitopes and are then separated from unbound library members using affinity-coupled magnetic beads. The resulting bacterial pools are grown overnight, plasmids encoding the antibody-binding peptides are purified, and the peptide-encoding regions are PCR amplified and barcoded with well-specific PCR indices. Ninety-four samples are normalized, pooled together and sequenced via next-generation sequencing (NGS). The output of SERA is a set of approximately 1 million peptide sequences for each individual, representing their unique epitope repertoire. After SERA screening, we applied two complementary discovery tools, IMUNE and PIWAS, to identify antigens and epitopes involved in the SARS-CoV-2 immune response (Fig. 1).

### Characterization of SARS-CoV-2 proteome antigens and epitopes

**Fig. 2:**
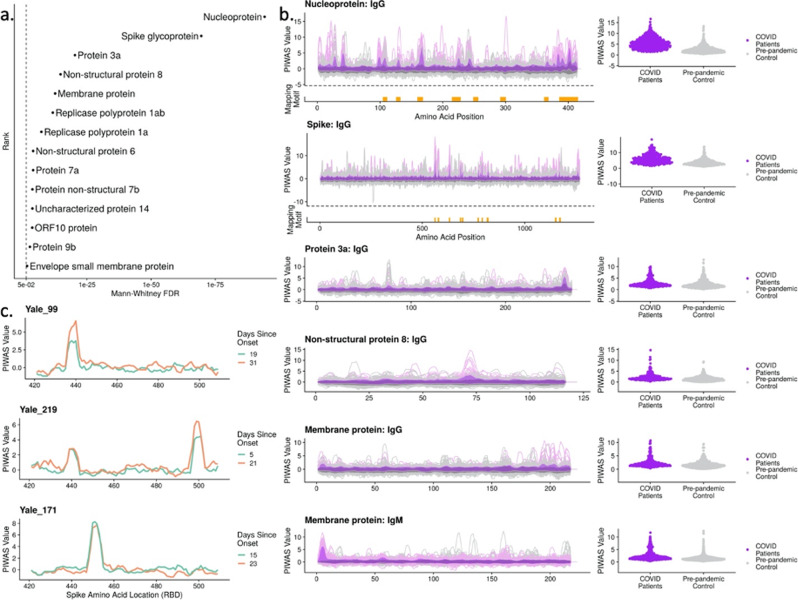
Bioinformatic analysis of SERA antibody repertoires identifies the antigens and epitopes involved in the SARS-CoV-2 immune response. **a** PIWAS statistical ranking of kmer enrichments across the SARS-CoV-2 proteome using the Mann–Whitney false-discovery rate (FDR). Multiple antigens in addition to spike and nucleoprotein showed significant enrichment for one or more epitopes. **b** PIWAS kmer enrichments from COVID-19 repertoires versus pre-pandemic controls across statistically significant antigens. PIWAS values = number of standard deviations above the mean of 1500 pre-pandemic controls. PIWAS tiling (left): Dark purple = average COVID-19 patient signal, light purple = 95th quantile band for COVID-19 patient signal, dark gray = average pre-pandemic control patient signal, light gray = 95th quantile band for pre-pandemic patient signal. PIWAS distribution (right): One point per patient of maximum PIWAS value across each protein. IMUNE motifs largely mapped to the same prominent epitopes that were identified by PIWAS. Epitopes on spike and nucleoprotein discovered by IMUNE are shown below each antigen (orange bars). All antigens were found to be statistically different from controls as shown in **a**. **c** Longitudinal samples from individual subjects enabled identification of RBD-specific signals that emerged over time but were not conserved across COVID-19 patients.

To establish an understanding of relevant SARS-CoV-2 antigens and epitopes, we analyzed the SARS-CoV-2 proteome with protein-based immunome wide association studies (PIWAS). Briefly, PIWAS identifies epitope signal in the context of an arbitrary proteome by tiling and smoothing kmer sequences across the entire proteome^28^. PIWAS derives power at both the cohort and single sample level through statistical comparisons to a large database of pre-pandemic controls. Using the reference SARS-CoV-2 proteome from Uniprot, we performed PIWAS of 579 COVID-19 samples compared to 497 pre-pandemic controls, with 1500 additional pre-pandemic controls serving as a normalization cohort. In addition to the established antigens spike and nucleoprotein, we observed highly significant signals for protein 3a, non-structural protein 8 (NSP-8), membrane protein, and replicase polyprotein 1ab (Fig. 2a). We further examined epitope-level signal for the top IgG and IgM antigens identified by PIWAS (Fig. 2b). Within spike and nucleoprotein, we observed multiple epitopes that are conserved across a large portion of the COVID-19 patient population. In contrast, epitope signals for protein 3a, NSP-8, and membrane protein (IgM) are largely characterized by a single, dominant epitope.

To validate the PIWAS epitopes identified in the random library screen, we constructed and screened a SARS-CoV-2 library consisting of 12mer peptides spanning the entire SARS-CoV-2 proteome with an 8 amino acid overlap (Methods). We show that the discovered epitope motifs that mapped to the SARS-CoV-2 proteome were also highly enriched in the SARS-CoV-2-specific peptide library (Supplementary Fig. 1 and Supplementary Data 2). Of the 22 PIWAS peaks with values over 2.5 73% also have a SARS-CoV-2-specific peptide library peak above 5000 reads, while 66% of the 22 SARS-CoV-2-specific peptide library peaks have a corresponding peak in PIWAS. One interesting example is, to the best of our knowledge, a novel spike epitope RSVASQSIIAYT that maps to the furin cleavage site (685–686) indicating that the antibody is likely reactive to the uncleaved version of the protein.

While the receptor binding domain (RBD) of spike is highly important in host infection by the virus, we observe no conserved linear epitope signal against this region of spike (amino acids 331–524). Instead, we observe private spike epitopes in a subset of patients in our cohorts (Fig. 2c and Supplementary Fig. 2). We highlight patients with epitopes observed in multiple longitudinal draws, to decrease the likelihood of false positive signal.

### Unbiased, proteome-independent epitope analysis

The IMUNE algorithm identified mapping and non-mapping epitope motifs that were highly enriched in COVID-19 repertoires (Methods)^27^. Linear epitopes identified by IMUNE largely overlapped with those identified by PIWAS (Fig. 2). The IgG linear motifs mapped to epitopes on spike glycoprotein (*n* = 10), nucleoprotein (*n* = 8) and NSP-8 (*n* = 2). IgM linear motifs mapped to a single epitope at the furin cleavage site on spike glycoprotein that was also a target for IgG antibodies, as well as one epitope on the SARS-CoV-2 membrane protein. Of the conserved, immunodominant epitope motifs identified by IMUNE, 27 of 45 IgG (60%) and 9 of 14 IgM (64%) did not directly map to the SARS-CoV-2 proteome. The identified motifs are highly specific to the COVID-19 patient population, with each individual motif present in ≤2% of 1500 pre-pandemic samples (Table 1—Test Set). Additionally, these motifs showed similar sensitivities in the SARS-CoV-2 training and test cohorts (Supplementary Data 3—average difference in motif sensitivity between training and testing sets of 4%, range 1–14%). We have observed from studies with monoclonal antibodies that non-mapping motifs represent mimotopes of both linear and structural epitopes.

**Fig. 3:**
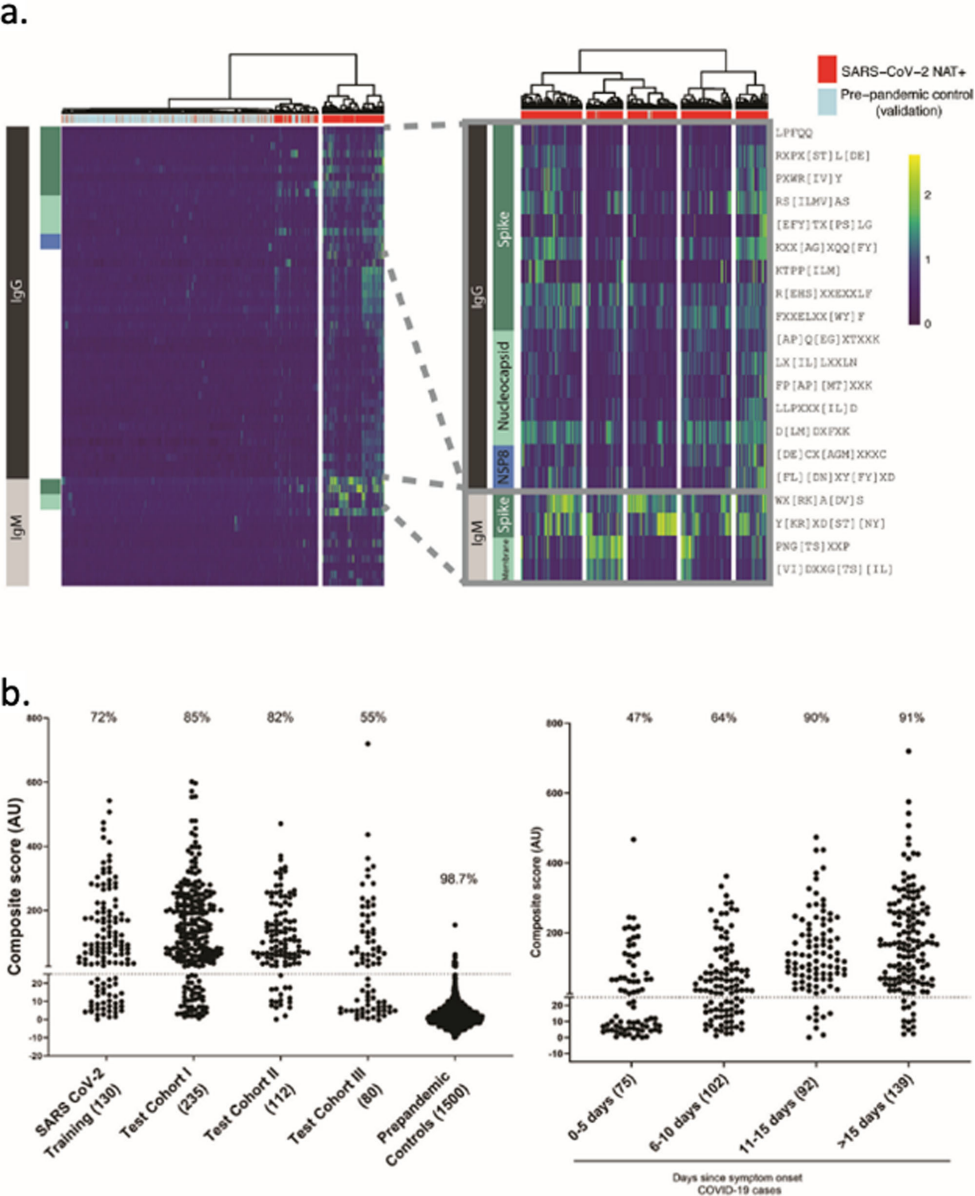
IMUNE-based discovery of IgG and IgM motifs in the SARS-CoV-2 humoral immune response. **a** Heatmap of IgG and IgM motif log-enrichment values for 579 COVID-19 samples and 1500 pre-pandemic controls. Inset highlights motifs with linear epitope maps to SARS-CoV-2. **b** Sensitivity and specificity of the SARS-CoV-2 IgG/IgM diagnostic classifier in NAT + subjects and pre-pandemic controls. Z-scores for each motif were summed to generate an IgG/IgM composite score. The maximum value for the IgG or IgM for each sample i shown. Samples above a cutoff of 25 are classified as positive. The sensitivity or specificity of the SERA panels for all COVID-19 cohorts and controls is shown above each column.

Motifs were selected for inclusion in the SARS-CoV-2 epitope map if they demonstrated a specificity of at least 98% in 497 pre-pandemic controls (Methods). The resulting SARS-CoV-2 panel of 45 IgG and 14 IgM motifs was compiled into a semi-quantitative epitope map, enabling visualization of motif enrichment for all evaluated COVID-19 and control samples (Fig. 3a). We observed that an unlabeled, hierarchical clustering of samples based on these motif enrichments largely separates pre-pandemic control samples from COVID-19 patients. Focusing on those motifs with linear hits to SARS-CoV-2, we further observed sub-clusters of patients with reactivity to specific isotypes and antigens, from left-to-right: spike IgG + IgM, spike and membrane IgM, spike IgM, nucleoprotein IgG, and broadly reactive.

### SARS-CoV-2 diagnostic panels can classify NAT + samples with sensitivity comparable to ELISA

To develop a SARS-CoV-2 diagnostic panel, we selected a subset of peptide motifs that, in the training cohort, either exhibited high sensitivity and specificity or improved the breadth of coverage. We normalized and summed motif enrichments to generate a composite score and compared sub-panels to identify the panel with the maximal diagnostic performance on the training cohort (Methods) (Table 1). A composite score of ≥25 was set as a cutoff for both IgG and IgM panels to obtain a specificity of ≥99% on the pre-pandemic training controls (Table 1). The panel performance was evaluated on a test cohort of 427 COVID-19 samples that were confirmed positive by NAT (Table 1, testing cohorts I-III). The classifier with the best overall performance is shown in Fig. 3b. The sensitivity varied between 54 and 82% across the NAT + cases from different cohorts, primarily based on the timing of blood collection relative to symptom onset for each cohort. A specificity of 99.3% for IgG and 99.1% for IgM was achieved on a test set of 1500 pre-pandemic repertoires that were tested for acute illness. Combining the IgG and IgM panels into a single test resulted in a panel specificity of 98.7%. Notably, no pre-pandemic samples were co-positive for IgG and M, thus the specificity for subjects that were positive for both IgG and IgM was 100% in the test control set. Forty-two percent of all tested COVID-19 samples met these criteria.

We plotted the SERA scores for samples from cohorts I and II, where timing of the blood draw relative to date of symptom onset was provided (Fig. 3b). The panel exhibited a sensitivity of 47% at 0–5 days after symptom onset, 64% at 5–10 days and ≥90% at >10 days post symptom onset. Where predicate SARS-CoV-2 ELISA (enzyme-linked immunosorbent assay) results were available, we compared performance relative to SERA in SARS-CoV-2 NAT + samples. Overall, SERA IgG and IgM panels together demonstrated similar sensitivity to three different ELISAs in current use (95% CI, Wilson score) (Table 2).

**Table 2:** SERA SARS-CoV-2 IgG or IgM panel sensitivity as compared with various ELISAs in NAT positive subjects.

Cohort	Serological test	*n*	SERA sensitivity (% 95% CI)	ELISA sensitivity (% 95% CI)
Yale	Spike S1 IgG and IgM ELISA	315	77 [71.8–81.1]	79 [73.8–82.9]
LabCorp	EuroImun S1 ELISA (IgG)	235	82 [76.7–86.5]	75 [69.4–80.4]
LabCorp	N Antigen ELISA (IgG)	235	82 [76.7–86.5]	86 [80.4–89.4]
SBCH	Spike S1 IgG and IgM ELISA	82	52 [41.8–62.9]	48 [37.1–58.2]
SBCH	Nucleoprotein IgG and IgM ELISA	82	52 [41.8–62.9]	50 [39.4–60.6]

### Motifs and epitopes associated with neutralization titer and disease severity

For each COVID-19 motif that we identified, we evaluated the relationship between the motif enrichment and SARS-CoV-2 neutralization titer for a subset of cases. We calculated the polyserial correlation for each motif and identified 13 motifs with a positive correlation (>0.2) with neutralization titer (Supplementary Fig. 3), with four of the motifs (FP[AP][MT]XXK, AXX[MS]RKP, D[LMY]SGI, and YWXYFXK) statistically significant after false-discovery rate adjustment (FDR < 0.1). The motif FP[AP][MT]XXK maps to nucleoprotein and therefore may simply correlate with neutralization or overall antibody titer. D[LMY]SGI is a linear mimotope of DISGI of spike glycoprotein (1168–1172), while the other two motifs are mimotopes that do not map directly to SARS-CoV-2 proteins.

**Fig. 4:**
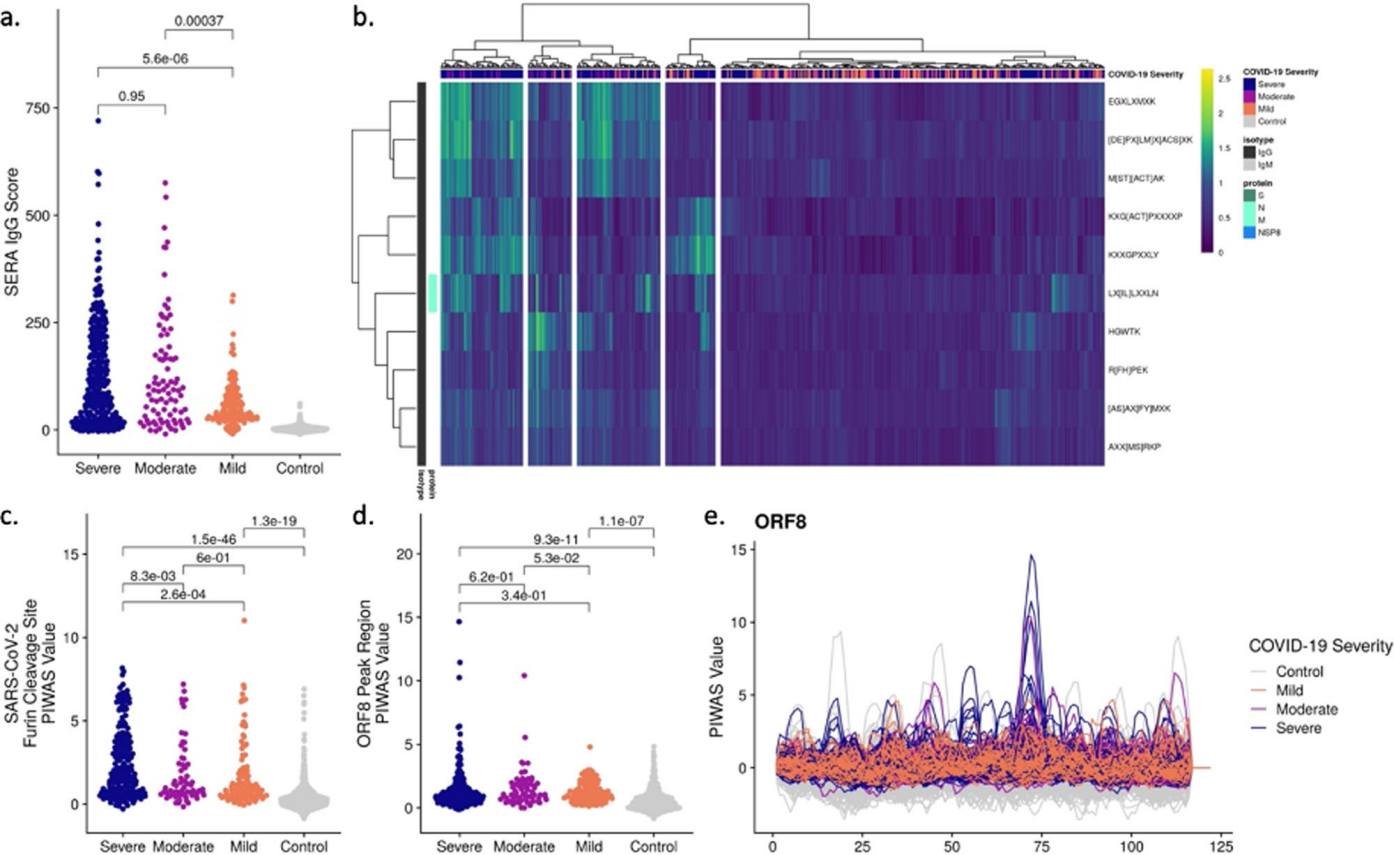
Significantly different epitope signals are observed in mild, moderate, and severe cases of COVID-19. **a** Comparison of SERA total IgG motif panel scores for severe (*n* = 447), moderate (*n* = 134), and mild (*n* = 170) cases based on motifs used in the diagnostic panel (Fig. 3). Colors indicate disease severity. **b** Severe, moderate, and mild cases of SARS-CoV-2 are clustered based on log-enrichment of the top 10 motifs identified by a t-test comparison of severe and mild patients. **c** Distribution of PIWAS values at SARS-CoV-2 furin cleavage site for severe, moderate, and mild cases. **d** Distribution of PIWAS values for the peak epitope in ORF8 for severe, moderate, and mild cases. **e** PIWAS tiling of individual samples on the entire ORF8 sequence. All p-values were calculated using outlier sum statistical test.

Based on prior studies that described subjects with severe disease possessing a stronger and, perhaps, earlier humoral IgG response in both spike and nucleoprotein relative to subjects with mild disease^29,30^, we examined differences in epitope prevalence based on disease severity. We compared the SERA IgG panel score (developed to distinguish COVID-19 patients from pre-pandemic controls) across the spectrum of severities present in our population (Fig. 4a). We observed a significant elevation of the panel score in patients with severe or moderate disease compared to their mild disease counterparts.

To understand the specific epitopes driving the severity delineation, we evaluated the relationship between COVID-19 severity and IgG motif enrichment. An FDR-adjusted t-test was used to identify motifs that were statistically significantly enriched in severe compared to asymptomatic/mild cases as well as the alternative hypothesis of motifs significantly enriched in asymptomatic/mild compared to severe cases (Supplementary Fig. 4). While we did not identify any motifs that were significantly enriched in mild disease, 48 of 59 motifs were statistically enriched in severe disease (FDR < 0.05).

We then selected the 10 motifs with the most significant t-test p-value when comparing severe and mild disease (Fig. 4b). We observe a potential confounding of days since onset of symptoms with the SERA IgG score (Supplementary Fig. 5). All 10 motifs were identified in the IgG screen and 9 out of 10 motifs did not possess a linear map to SARS-CoV-2. In the hierarchical clustering of samples, we observe subsets of severe patients with preferential enrichment for differing motifs. After splitting our data into 2/3 training and 1/3 testing cohorts, we built a simple LASSO (least absolute shrinkage and selection operator) model to classify moderate/severe from mild disease, and observed encouraging performance (training AUC 0.92, testing AUC 0.9, Supplementary Fig. 5).

One of the distinguishing features of the SARS-CoV-2 coronavirus is the acquisition of polybasic residues (RRAR) at the cleavage site of the S1/S2 boundary. Cleavage of spike protein at this site facilitates viral membrane fusion^31,32^. It has been proposed that this novel sequence enables the virus to take advantage of host proteases, such as furin, that cleave proteins with this recognition sequence, thereby increasing the potential tropism of the virus relative to other coronaviruses^31,33^. We asked if this site elicited an immune response, and if so, was it seen differentially in subjects with different disease severity. In the spike epitope map, signal at this sequence location is both prominent and prevalent in the cohorts—120 out of 385, or 31% of subjects, had epitope signals >99% of that seen in controls. We also determined that the site elicited a statistically significantly stronger immune response in subjects with severe disease relative to subjects with mild or moderate disease (Fig. 4c). Specifically, 39%, 23%, and 20% of severe, moderate, and mild cases, respectively, had strong epitope signals greater than 99% of that in the controls.

In addition to spike and nucleoprotein, a robust immune response has been described against the ORF8 protein^34^. Several reports have described a variant of SARS-CoV-2 with a 382-nucleotide deletion in ORF7b and ORF8 as well as an association of the deletion with a milder disease course^35^. While we do not have genotype information for all strains, based on the GISAID database we assume that most of the samples in our cohorts do not have this deletion. To explore the possible association of immune response with disease severity, we analyzed the PIWAS signal against ORF8, which encompasses most of the 382-nucleotide deletion. While there appear to be more extremely high signals in severe cases, using an outlier sum statistic, the PIWAS signal in ORF8 does not reach statistical significance in severe cases relative to mild and moderate cases (Fig. 4c, e).

### PIWAS prediction of antibody cross-reactivity to other coronaviruses

**Fig. 5:**
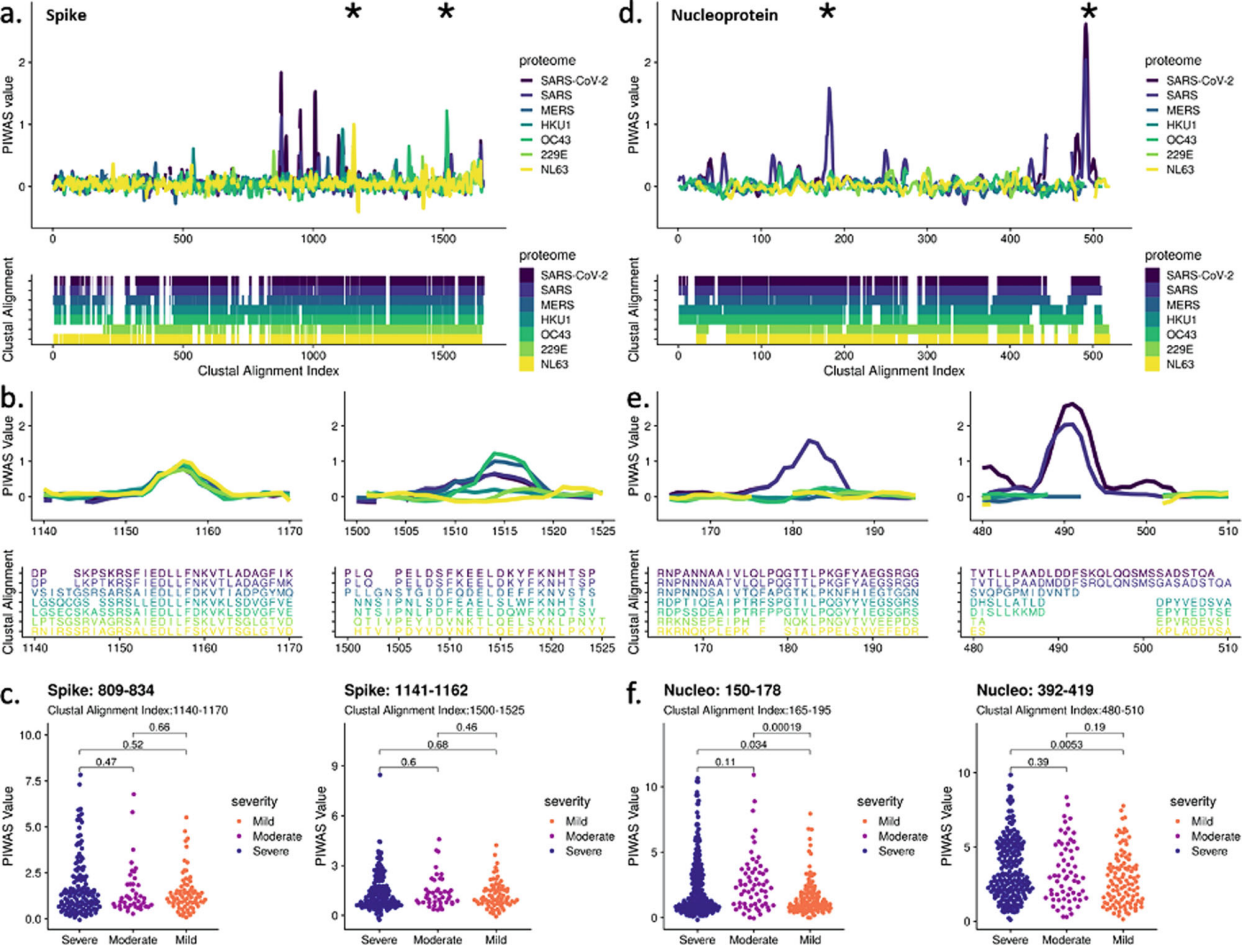
Cross-reactivity analysis across coronaviruses reveals shared epitopes and epitopes specific to SARS-CoV-2. PIWAS was performed using COVID-19 samples against various coronavirus proteomes including SARS-CoV-2, SARS, MERS, and the common hCoVs HKU1, OC43, 229E, and NL63. PIWAS tilings for **a** spike and **d** nucleoprotein revealed regions of cross-reactivity as well as epitopes only observed against SARS-CoV-2. Clustal multiple sequence alignments were performed and visualized below to depict sequence similarity and divergence. Distinct epitopes from **b** spike and **e** nucleoprotein showcase PIWAS values across the coronaviruses with corresponding clustal alignment sequences below. Epitope locations are denoted with asterisks in **a** and **d**. Distribution of PIWAS values at epitopes from spike **c** and nucleoprotein **f** for severe (*n* = 447), moderate (*n* = 134), and mild (*n* = 170) cases, with *p*-values from Wilcoxon-rank sum test.

We next investigated SARS-CoV-2 epitopes that may cross-react with other coronaviruses as previous exposure to coronaviruses could have protective or even deleterious effects on symptoms^6,7^. To identify potential cross-reactive epitopes, we performed PIWAS using the epitope repertoires from COVID-19 samples against various coronavirus proteomes, including SARS-CoV-2, SARS-CoV (SARS), MERS-CoV (MERS), and the four common human coronaviruses (hCoVs) HKU1, OC43, 229E, and NL63. Analysis of average PIWAS values for spike glycoprotein across coronaviruses revealed epitopes that were conserved in many coronaviruses as well as epitopes that were specific to SARS-CoV-2 (Fig. 5a). We identified 10 epitopes enriched against the SARS-CoV-2 proteome (average PIWAS > 0.5), two and one of which overlapped with OC43 and NL63 epitopes, respectively. For example, the region corresponding to spike 809–834 in SARS-CoV-2 (alignment indices 1140–1170) contained an epitope that was observed against all coronaviruses analyzed (Fig. 5b). However, at spike 1141–1162 in SARS-CoV-2 (alignment indices 1500–1525) an epitope was observed only against SARS-CoV-2, SARS, MERS, and OC43 proteomes, with OC43 exhibiting the highest average PIWAS value. After evaluating enrichment for these cross-reactive spike epitopes in COVID-19 cases with different disease severity, we found there was no statistical difference between severe, moderate, and mild cases (Fig. 5c).

In contrast to spike, nucleoprotein exclusively contained epitopes specific to SARS-CoV-2 and SARS, with 4 epitopes against the SARS-CoV-2 proteome (Fig. 5d). Strong epitopes were observed against SARS-CoV-2 at regions 150–178 (alignment indices 165–195) and 392–419 (alignment indices 480–510) with no signal observed against hCoVs (Fig. 5e). We determined that these nucleoprotein epitopes were significantly enriched in severe and/or moderate cases compared to mild cases (Fig. 5f).

### Epitope signal in mutated SARS-CoV-2 strains

**Fig. 6:**
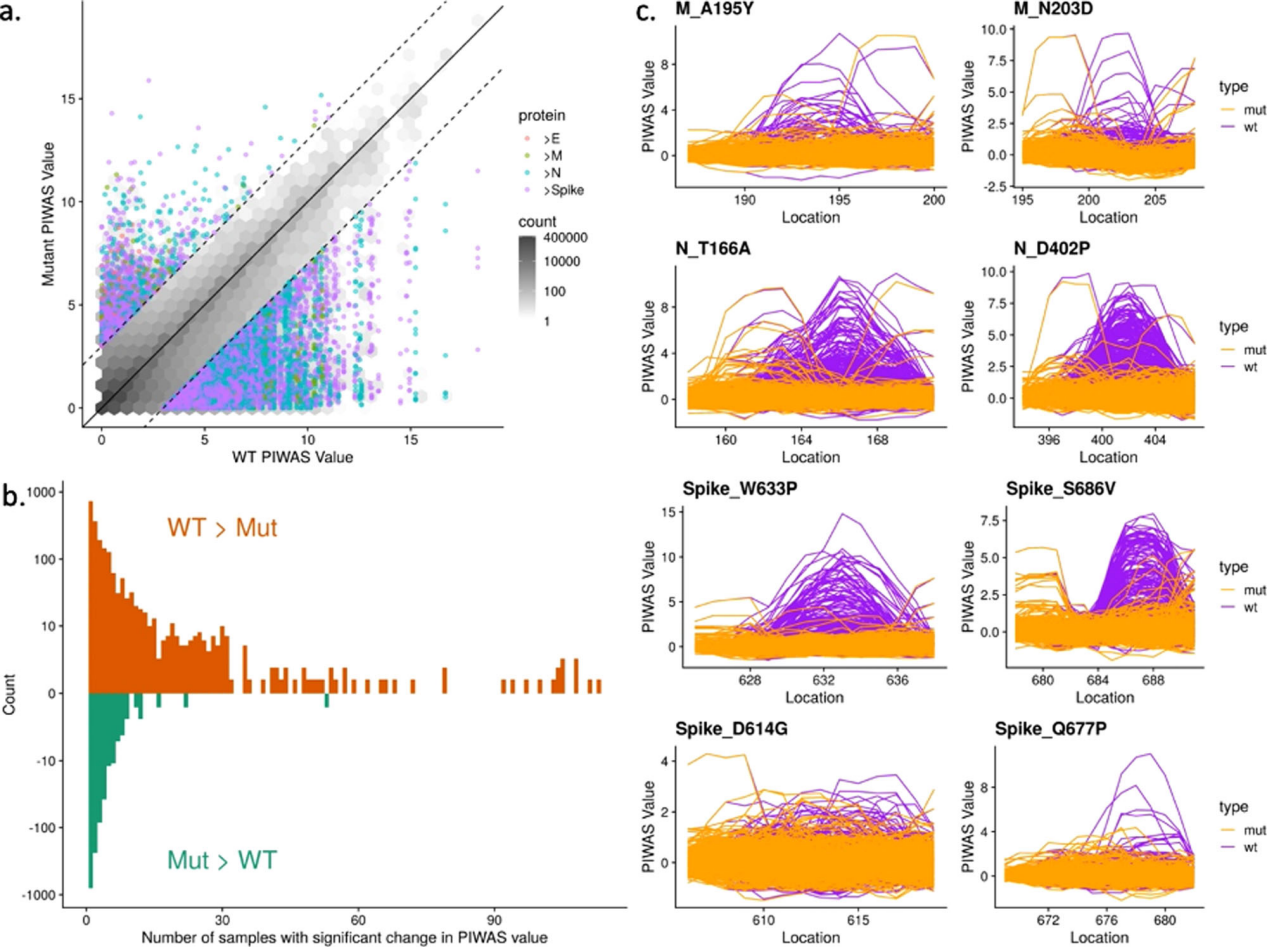
Mutations to SARS-CoV-2 are biased towards decreasing immune epitope response. 21,127 distinct amino acid mutations in spike glycoprotein, nucleoprotein, envelope protein, and membrane protein in SARS-CoV-2 strains were identified from sequencing data of 96,437 genomes from GISAID. **a** For each mutation, the PIWAS value of the wild-type (WT) sequence was compared to the PIWAS value for the mutated strain (mut). **b** Mutations conferring a significant PIWAS value change (|PIWAS_WT_-PIWAS_mut_ | > 3) for each COVID-19 sample were identified. For each mutation, the number of patients with a significant difference was counted. **c** Exemplary mutations that yielded a decrease in PIWAS values are shown for membrane protein (top row), nucleoprotein (middle row), and spike (bottom row). No significant immune signal is seen at location 614 of spike, for either the wild-type or the D614G variant. The Q677P mutation in spike resulted in a loss of signal at that epitope.

To study the possible effects of known mutations to the SARS-CoV-2 virus on antibody response, we leveraged the ability of PIWAS to interrogate the SERA database with any sequence of interest. In the 96,437 sequenced strains from GISAID, we enumerated 21,127 distinct amino acid mutations to spike glycoprotein, nucleoprotein, envelope protein, and membrane protein^36,37^. For each mutation, we compared epitope signal against the wild-type (WT) and mutant position in every COVID-19 specimen. We observed a bias towards mutations yielding a decreased PIWAS signal relative to WT (Fig. 6a). A subset of these mutations yielded decreased signal across a large number of COVID-19 patients (Fig. 6b). To assess the significance of this decreased epitope signal, we in silico randomly mutated amino acids throughout the same protein sequences as a null distribution. The Kolmogorov-Smirnov test comparing the observed and null distributions was highly significant (p = 3e-11), indicating that the bias towards mutants that generate a decreased epitope signal exceeds that which would be explained purely due to chance (Supplementary Fig. 6). For membrane protein, nucleoprotein, and spike glycoprotein, we highlight exemplar mutations, which resulted in decreased epitope signal across a large number of patients (Fig. 6c) and, in the case of spike glycoprotein, are on the surface of the protein according to the crystal structures considered in this paper^31,32^. In contrast, the dominant spike glycoprotein D614G exhibits no epitope signal for either the wild-type or mutant strains (Fig. 6d). We did observe a loss in epitope signal at Q677P, which has recently arisen in several lineages in the United States^38^.

## Discussion

While conventional serology is a cornerstone of infectious disease diagnosis, the COVID-19 pandemic has raised many questions not answered by these testing modalities alone. Here we have shown that high-content random bacterial peptide display library screening using SERA provides a tool to broadly and deeply probe individual antibody repertoires. These profiles, both individually and in the aggregate, can yield insights into disease severity, immunity, cross-reactivity to other coronaviruses (including SARS-CoV-2 mutant strains), and autoimmune sequelae.

By taking a focused, proteome-constrained approach to identifying signal against the SARS-CoV-2 proteome using both a library of random peptides, and a focused library of peptides from the proteome itself, we both reiterate the established immunological relevance of spike and nucleoprotein as well as identify less described signals against protein 3a and NSP-8. Epitope-level characterization of these antigens highlights particularly immunogenic epitopes within each protein, which might serve as targets in the development of vaccines and therapeutics. In particular, we identified strong epitopes in nucleoprotein including at amino acids 158–172 and the C-terminal domain 380–419, as well as epitopes in spike glycoprotein including at amino acids 555–572, 810–828, and 1145–1159, consistent with previous studies^4,24,39^. Additionally, we highlight novel and less-studied epitopes, including a dominant IgM epitope from membrane protein (amino acids 1–12), which could provide utility in early diagnostics. While we did not observe spike RBD epitopes that were conserved across the patient cohort, we found compelling examples of private RBD epitopes. The lack of linear epitopes towards spike RBD is unsurprising given the complex structural nature of spike, with many strands running in parallel likely yielding an abundance of structural mimotopes (also reflected in the quantity of non-mapping motifs in our diagnostic panel).

Leveraging our database of thousands of pre-pandemic repertoires collected from healthy individuals as well as people with infections, autoimmune diseases, and cancer across all age groups and geographies, we were able to assess the specificity of the SARS-CoV-2 antibody response and identify a panel of epitopes that could distinguish COVID-19 cases from controls with accuracy similar to conventional serological testing. An important distinction between SERA and non-random peptide array-based analysis is that more than half of the epitope motifs identified by the IMUNE algorithm do not directly map to the SARS-CoV-2 proteome but improve the performance of both diagnostic and severity classifiers. These peptides represent potentially novel biomarkers that also could serve as reagents to isolate antibodies or B cells to study their functions or for therapeutic development pipelines. Consistent with previous studies, we find that the humoral immune response against SARS-CoV-2 is stronger in severe and moderate disease relative to mild disease^4,40,41^. This finding is consistent with a general pattern of disease associated with immunopathology in COVID-19. We also identified specific epitope profiles that correlate with disease severity and combined these epitopes into a preliminary disease severity classification model. To further validate these findings, we would require a separate validation cohort of patients, which span mild and severe disease states. Ideally, future studies might evaluate these profiles longitudinally and early in disease to ascertain if they may also have prognostic significance on severity of disease. While many discovered motifs were statistically enriched in severe disease, those that are conserved across all disease states may be considered for potential targeting by therapies and vaccines. Importantly, many of our disease severity analyses are potentially confounded by the number of days since onset of symptoms in the COVID-19 subpopulations, partially due to the challenge of both identifying disease onset in mild patients and collecting samples from non-hospitalized patients.

Milder disease has been described in subjects with the 382-ORF8 deletion variant, and the ORF8 protein has been noted to be associated with strong humoral response^35,42^. In our study, we also see significant response relative to a pre-pandemic cohort in ORF8. While a few epitopes appear quite strong in some individuals in ORF8 with severe disease, the overall signal across the antigen was not seen to be statistically significant in mild versus severe disease. Specific, strong epitope signals in ORF8 could be postulated to contribute to severe disease through a variety of mechanisms, but this would also need to be explored through further epidemiological and experimental analysis.

The S1/S2 cleavage site was found to be both prominent and more prevalent in severe disease relative to mild disease. This novel epitope was identified by PIWAS and IMUNE as well as the SARS-Cov-2-specific library. Interestingly, this epitope was not reported to be enriched using the virscan technology^24^. The increase in epitope signal at this site may be simply a reflection of severe disease, but could be considered somewhat unexpected if antibody binding prevents cleavage, as the site has been shown in animals to contribute to viral pathogenesis^43^. While SERA can be used to assess for autoantigens with PIWAS, assessment of autoantigens is beyond the scope of this manuscript. It should be noted though that the polybasic S1/S2 sequence (^682^RRARSVASQ) is shared uniquely with the human ENaC sodium channel (^201^RRARSVASS), which is also cleaved and activated by furin at this site, and has been postulated to have a role in the pathophysiology of SARS-CoV-2 infection^44^. Potential molecular mimicry at this site should thus be considered in light of the increasing recognition of the role of autoantigens in acute COVID-19 and MIS-C^45,46^.

By evaluating epitope signal in COVID-19 cases against common human coronavirus (hCoV) proteomes, we predicted prevalent cross-reactive epitopes particularly in the S2 domain of spike. Given the strength and prevalence of these cross-reactive epitopes, it is plausible that previous exposure to hCoVs contributed to these antibody responses, a boosting phenomenon recently described in COVID-19 cases^47^. In particular, the cross-reactive epitope at spike amino acids 809–834 near the fusion domain has been shown to elicit an antibody response in SARS-CoV-2 uninfected adolescents and adults^48^. Interestingly, antibodies targeting this epitope demonstrated neutralizing capacity using antibody depletion assays^39^. More broadly, the presence of spike-reactive T cells in healthy donors has been observed against SARS-CoV-2 as well as hCoVs 229E and OC43, primarily reactive towards the spike S2 domain^6^. While these findings suggest a role for cross-reactive epitopes in the response to SARS-CoV-2 infection, it is uncertain what impact pre-existing antibodies have towards protection, immunity, and disease progression. Recent studies suggest that pre-existing antibodies from hCoVs exist but are not associated with protection^47,49^. We observed that prevalent cross-reactive epitopes in spike were not associated with COVID-19 severity while multiple nucleoprotein epitopes specific to SARS-CoV-2 and SARS were significantly enriched in severe cases compared to mild. Notably, it has been shown that convalescent COVID-19 patients exhibited a shift in antibody response towards spike compared to a nucleoprotein-directed antibody response in deceased patients^3^. Given that cross-reactive epitopes were observed in spike, additional investigation will be critical towards understanding pre-existing antibody responses that may impact SARS-CoV-2 infection and COVID-19 progression.

While it has been shown that some mutations alter viral fitness and/or infectivity, some mutations may also decrease antibody binding or protection. Under this hypothesis, SARS-CoV-2 strains are undergoing selective pressure to evade antibody response, resulting in strains that may be less susceptible to clearance by those previously infected with wild-type SARS-CoV-2^50,51^. The public health consequences of epitope mutation are concerning, and further suggestive of the potential for the SARS-CoV-2 virus to periodically re-emerge and reinfect individuals with prior exposure. We have developed a tool that can analyze the effect of mutations on antibody binding to arbitrary linear epitopes from a proteome of interest. The decreased epitope signal in COVID-19 patients against mutant strains of SARS-CoV-2 compared to WT could be suggestive of evolutionary evasion of the antibody response^52–54^. This underscores the importance of monitoring epitope mutations to guide therapeutic and vaccine development efforts and focus on epitopes that are less susceptible to evasion, which would be more broadly cross-reactive and robust to evolutionary changes.

The dominant strains of SARS-CoV-2 that are now in circulation possesses the D614G mutation. Based on our data, neither the wild-type nor the mutant confer a strong linear epitope, consistent with observations that the mutation is most notable for its effect on the structure of spike^55^. The Q677P mutation, however, did result in a loss of epitope signal. This mutation is of interest given the proximity to the polybasic furin cleavage site, and it is thought that mutations near this site may confer an advantage in spread or transmission^38^.

We acknowledge various limitations with the SERA platform that impact this study. Much of this study has focused on dominant epitopes prevalent in COVID-19 cases, but many of the private epitopes not explicitly discussed here, particularly in spike RBD, are critical to fully understanding the protective antibody response and clinical outcomes. Moreover, there are clear limitations for probing the epitope repertoire with linear peptides, chiefly the challenges of identifying structural epitopes and the role of post-translational modifications such as glycans^56^. The discovery of epitopes using PIWAS is based on a normalization to a pre-pandemic database of individuals whose common coronavirus serology status is unknown. The population serostatus could thus bias both positive and negative findings for any specific epitope. This is mitigated by the strength and diversity of strong epitopes signals discovered, many of which have been identified in previous studies. In our analysis of mutational effects on immune evasion, we note that our use of the null distribution based on unbiased amino acid selection under-represents codon bias. For epitopes such as E484K that is thought to be part of a structural epitope, and that does not have a linear epitope signal seen in our assay, there is no signal for “evasion” to occur. Thus, our method is limited to regions where linear epitopes are present in “wild-type” sequences. Finally, while a random peptide library enables unique opportunities to identify structural mimics, much work remains in cataloging and mapping these mimics to their cognate antigens.

In summary, we present the application of SERA to assess SARS-CoV-2 seropositivity and to characterize a high-resolution map of motifs and epitopes in individuals and populations. We demonstrate the ability of the platform to assess disease severity, to compare in silico epitope response to multiple coronavirus strains, to assess potential immune escape at sites of variation, and to evaluate longitudinal changes in signal, all with one assay. The random nature of the libraries, the ability to identify non-mapping, highly specific disease epitopes, and to leverage quality-controlled reference data from a large pre-pandemic cohort all contribute to SERA’s ability to elucidate the humoral immune response in SARS-CoV-2 infection.

Our findings support those of other studies that find clear differences in the humoral response of individuals with different clinical severity and trajectories. While we may identify associations between high-resolution epitope and motif signals and disease severity, much work is required to establish functional or causal relationships. Examining and correlating epitopes to clinical efficacy in the context of vaccines and therapeutic antibodies will help to elucidate the connection between measured immune response and patient outcome.

Yet the epitope landscape can change, as it is already clear that coronaviruses mutate and SARS-CoV-2 is no exception. Potential changes in the infectivity of the virus in just this first year of the current health crisis underscore the need to track evolving immune responses and clinical features in populations world-wide^55,57–60^. We have demonstrated the ability to capture and query both past and present repertoires through analysis of pre-pandemic and current pandemic samples. Using SERA to observe longitudinal immune responses in the context of persistent symptoms or reinfection enables construction of a detailed picture of infection, immunity and disease in COVID-19. SERA’s ability to query against any variant or future emerging genomes can be used to support ongoing management of the current health crisis and future novel outbreaks.

## Methods

### Study design, statistics, and reproducibility

Sample sizes for COVID-19 cohorts were not calculated based on statistical methods and were based on the number of subjects and samples available from each study/cohort. Subject samples were stratified based on COVID-19 diagnosis and disease severity. Sample sizes for pre-pandemic controls were driven by statistical calculations for PIWAS analysis, and a limit of at least 500 control samples has been established. We used 1,500 control samples given the availability of additional pre-pandemic controls. Samples were obtained and analyzed from multiple cohorts to reduce any cohort-dependent effects.

Extensive internal validation for the SERA assay, including technical and biological variation, has been completed, including a precision study of COVID-19 serum samples with respect to SARS-CoV-2 epitope enrichment. Additionally, every 96-well plate of samples processed for this study contained healthy control run standards in replicate to assess and evaluate assay run reproducibility and possible batch effects. At the time of sample acquisition, samples were designated with generic sample IDs and scientists were unaware of sample cohort status during sample processing. The COVID-19 diagnostic panels developed in this study were trained on discovery cohorts and validated on independent testing cohorts. Other findings were not explicitly replicated.

### Biospecimens and cohorts

COVID-19 diagnosis was determined by positive PCR test and/or ELISA for SARS-CoV-2, and controls were restricted to samples collected prior to the SARS-CoV-2 outbreak (pre-pandemic). Sera or plasma from confirmed or suspected COVID-19 cases were acquired from Yale, Santa Barbara Cottage Hospital (SBCH), LabCorp, BioIVT and Blood Centers of America (BCA). Samples were de-identified prior to receipt at Serimmune. Santa Barbara Cottage Health IRB approved the sample collection for the SBCH cohort with human subject research exemption. All other samples included in this study were remnant de-identified samples. All samples and associated metadata are shown in Supplementary Data 1.

### Yale cohort

Patients admitted to the Yale New Haven Hospital (YNHH) were recruited to the Yale IMPACT study (Implementing Medical and Public Health Action against Coronavirus CT) after testing positive for SARS-CoV2 by qRT-PCR or after a suspected COVID-19 diagnosis followed by a positive SARS-CoV-2 serology test. Patients were identified through screening of EMR records for potential enrollment with no self-selection. Informed consent was obtained by trained staff and sample collection commenced immediately upon study enrollment. Health care workers were recruited as part of a longitudinal monitoring study. Subjects with a positive SARS-CoV-2 qRT-PCR test of positive serology were included in this study. COVID-19 cases were classified as mild if patients were not hospitalized, moderate if hospitalized, and severe if on high-flow nasal canula, bilevel positive airway pressure (BiPAP) or other non-invasive ventilation, intubated or died from COVID-19. The Yale IMPACT biorepository study was approved by Yale Human Research Protection Program Institutional Review Boards (protocol ID 2000027690) and participation in the study was voluntary.

### SBCH cohort

Biobanked sera or plasma from individuals that previously tested positive for COVID-19 were provided by the SBCH Biobank. Clinical data, including age, sex, and disease severity were obtained by SBCH staff for inclusion in the biobank. Specimens were collected from both inpatient and ambulatory settings and were coded as asymptomatic, mild/moderate if the subject had symptoms consistent with COVID-19, or severe if the individual required admission to the ICU for symptoms. Participation in these studies was voluntary and the study protocol was approved by the SBCH Institutional Review Board.

### LabCorp cohort

The majority of samples were remnant sera from acutely ill, ICU hospitalized, PCR confirmed COVID-19 cases with high IL-6 test results (*n* = 235). These cases were classified as severe disease. An additional ten suspected COVID-19 cases were from individuals with mild symptoms. A subset of these had serological evidence of infection by anti-RBD ELISA and/or neutralization assay data.

### BioIVT cohort

Remnant serum samples with serological evidence of infection by a positive Epitope EDI IgG test (*n* = 20) or a positive NAT test (*n* = 1) were purchased from BioIVT. A subset had disease severity characterization provided by the vendor (Supplementary Data 1).

### BCA cohort

Plasma samples were collected from healthy blood donors in New York during the period of March–July of 2020 as part of a collaboration with The Blood Centers of America (BCA). Two samples included in the study were collected from COVID-19 plasma donors with confirmed disease. Suspected COVID-19 cases included in the study had serological evidence of infection based on a positive SERA IgG or IgM result that was subsequently confirmed by S1 spike and nucleoprotein ELISA IgG in the majority of cases. Cases from healthy donors were classified as mild disease.

### SERA serum screening

A detailed description of the SERA assay has been published^26^. For this study, serum or plasma was incubated with a fully random 12-mer bacterial display peptide library (1 × 10^10^ diversity, 10-fold oversampled) at a 1:25 dilution in a 96-well, deep well plate format. Antibody-bound bacterial clones were selected with 50 µL Protein A/G Sera-Mag SpeedBeads (GE Life Sciences, cat#17152104010350) (IgG) or by incubation with a biotinylated anti-human IgM antibody (Jackson ImmunoResearch, cat# 709-066-073) final assay dilution 1:100, followed by a second incubation with 50 µl Dynabead MyOne Streptavidin T1 conjugated magnetic beads (IgM) (Thermo-Fisher 65602). The selected bacterial pools were resuspended in growth media and incubated at 37 °C shaking overnight at 300 RPM to propagate the bacteria. Plasmid purification, PCR amplification of peptide-encoding DNA, barcoding with well-specific indices was performed as described^26^. Samples were normalized to a final concentration of 4 nM for each pool and run on the Illumina NextSeq500. Every 96-well plate of samples processed for this study contained healthy control run standards to assess and evaluate assay reproducibility and possible batch effects.

### Spike S1 and nucleoprotein ELISA

The SARS-CoV-2 spike S1 and N antigen ELISA data were provided by Yale and LabCorp. spike S1 and nucleoprotein ELISAs on the SBCH COVID-19 samples were performed in house using recombinant proteins (ACRO Biosystems, S1N-C52H3 and NUN-C5227, respectively). A cutoff value for positivity was established using 3 times the standard deviation of 502 pre-pandemic controls for the IgG and 82 pre-pandemic controls for IgM assays. Briefly, plates (Nunc MaxiSorp) were coated with recombinant proteins, 0.5 µg/mL for IgG and 1 µg/mL for IgM at 4 °C overnight. After washing, plates were blocked with PBS (phosphate buffered saline) containing 5% non-fat milk for 2 h at room temperature. Plates were then incubated with serum diluted 1/250 in blocking buffer for 1 h at room temperature. Plates were washed, then incubated with horseradish peroxidase (HRP) conjugated goat anti-human IgG or HRP-donkey anti-human IgM (Jackson ImmunoResearch) secondary antibody diluted 1/10,000 in blocking buffer for 1 h at room temperature. After washing, the reaction was developed with 3,3′,5,5′-teramethylbenzidine substrate solution (ThermoFisher) for 15 min and stopped with 1 M HCL. The absorbance was measured on a Tecan Spectrafluor plus plate reader at 450 nm.

### Cell lines and virus

VeroE6 kidney epithelial cells were cultured in Dulbecco’s Modified Eagle Medium (DMEM) supplemented with 1% sodium pyruvate (NEAA) and 5% fetal bovine serum (FBS) at 37 °C and 5% CO_2_. The cell line was obtained from the ATCC and has been tested negative for contamination with mycoplasma. SARS-CoV-2, strain USA-WA1/2020, was obtained from BEI Resources (#NR-52281) and was amplified in VeroE6 cells. Cells were infected at a MOI 0.01 for four three days to generate a working stock and after incubation the supernatant was clarified by centrifugation (450 × *g* × 5 min) and filtered through a 0.45-micron filter. The pelleted virus was then resuspended in PBS then aliquoted for storage at −80 °C. Viral titers were measured by standard plaque assay using Vero E6 cells. Briefly, 300 µl of serial fold virus dilutions were used to infect Vero E6 cells in MEM supplemented NaHCO_3_, 4% FBS 0.6% Avicel RC-581. Plaques were resolved at 48 h post infection by fixing in 10% formaldehyde for 1 h followed by with 0.5% crystal violet in 20% ethanol staining. Plates were rinsed in water to plaques enumeration. All experiments were performed in a biosafety level 3 with the Yale Environmental Health and Safety office approval.

### Neutralization assay

Patient and healthy donor sera were isolated as before and then heat treated for 30 m at 56 °C. Sixfold serially diluted plasma, from 1:3 to 1:2430 were incubated with SARS-CoV-2 for 1 h at 37 °C. The mixture was subsequently incubated with VeroE6 cells in a 6-well plate for 1 h, for adsorption. Then, cells were overlayed with MEM supplemented NaHCO_3_, 4% FBS 0.6% Avicel mixture. Plaques were resolved at 40 h post infection by fixing in 10% formaldehyde for 1 h followed by staining in 0.5% crystal violet. All experiments were performed in parallel with negative controls sera, at an established viral concentration to generate 60–120 plaques/well.

### PIWAS analysis

We applied the published PIWAS method^28^ to identify antigen and epitope signals against the Uniprot reference SARS-CoV-2 proteome (UP000464024)^61^. For each sample, approximately 1–3 million 12mers are obtained from the SERA assay and these are decomposed into constituent 5 and 6 mers. Enrichment scores for each kmer are calculated by dividing the number of unique 12 mers containing the kmer divided by the number of expected kmer reads for the sample, based on amino acid proportions in the sample. The PIWAS analysis was run on the IgG SERA data with a single sample per COVID-19 patient (for a total of 579 patients) versus 497 discovery pre-pandemic controls, and the 1500 validation controls used as the normalization cohort. Additional parameters include: a smoothing window size of 5 5 mers and 5 6 mers; z-score normalization of kmer enrichments; maximum peak value; and generation of epitope-level tiling data. Antigens were ranked using the Mann–Whitney U false-discovery rate, following the hypotheses of conserved epitopes in the context of infectious disease. For top antigens, tiling data was generated for every case and control sample. 95th quantile bands were calculated based on each population separately. The most prominent RBD epitopes were identified in COVID-19 patients with draws from at least 2 timepoints and a PIWAS value of at least 6 occurring between the 319th and 541st amino acids.

### IMUNE-based motif discovery

Peptide motifs representing epitopes or mimotopes of SARS CoV-2-specific antibodies were discovered using the IMUNE algorithm^27^. A total of 164 antibody repertoires from 98 hospitalized subjects from the Yale IMPACT study (Table 1) were used for motif discovery. The majority of subjects were confirmed SARS CoV-2 positive by NAT. IMUNE compared ~30 disease repertoires with ~30 pre-pandemic controls and identified peptide patterns that were statistically enriched (*p*-value ≤ 0.01) in ≥25% of disease and absent from 100% of controls. Multiple assessments were run with different subsets of cases and controls both for IgG and IgM. Peptide patterns identified by IMUNE were clustered using a point accepted mutation 30 (PAM30) matrix and combined into motifs. The output of IMUNE included hundreds of candidate IgG and IgM motifs. A motif was classified as positive in a given sample if the enrichment was ≥4 times the standard deviation above the mean of the training control set (Table 1). The candidate motifs were further refined based on at least 98% specificity. The final set of motifs was validated for sensitivity and specificity on an additional 1500 pre-pandemic controls and 406 unique confirmed COVID-19 cases from four separate cohorts (test cohorts I-IV, Table 1).

### Development of a diagnostic classifier for COVID-19

To generate a diagnostic score that classified subjects as serologically positive for antibodies to COVID-19, motif enrichment values were normalized using the mean and standard deviation of enrichments within the training set of pre-pandemic control repertoires (Table 1). Individual SARS-CoV-2 motif normalized “*z*-scores” were then summed to obtain a composite score for each sample. A composite score of 25 was established as a cutoff for positivity for each panel to obtain a specificity of >99% on the pre-pandemic training controls.

### Analysis of motif enrichment and neutralization titer

For samples where the SARS-CoV-2 neutralization titer was measured (*n* = 44), we evaluated the relationship of neutralization titer with COVID-19 motif enrichment. For each motif that we identified, we calculated the polyserial correlation between neutralization titer (1:1000, 1:100, 1:10, or none) and motif enrichment with a false-discovery rate adjustment.

### Mild versus severe disease analysis

For samples where clinical severity was known, we compared SERA IgG panel scores using the outlier sum statistic^28,62^. Using a *t*-test, we compared enrichments for all IgG and IgM motifs between the severe and mild populations and identified motifs that were statistically significant (FDR < 0.05). The 10 most significant motifs were highlighted and hierarchically clustered (Euclidean distance, Ward clustering^63^). Severity based on PIWAS signal against the furin cleavage and ORF8 regions was similarly compared using the outlier sum statistic.

### Common coronavirus analysis

We identified Uniprot reference proteomes for the four common human coronaviruses [OC43 (UP00007552), HKU1 (UP000122230), 229E (UP0006716), and NL63 (UP000145724)] and more severe strains [SARS (UP000000354) and MERS(UP000171868)]^61^. For each proteome, we ran a PIWAS with the same parameters as the SARS-CoV-2 PIWAS (above). For spike glycoprotein and nucleoprotein, we averaged PIWAS tiling values for the COVID-19 cohort across each proteome. A multiple sequence alignment of all these coronavirus sequences was performed using Clustal Omega^64^. Using this alignment index, we identified regions of divergent and convergent signal across the coronavirus proteomes in the COVID-19 population. For regions of interest, we calculated the significance of differences in patient severity using the Wilcoxon-rank sum test.

### GISAID originating laboratories

Proteome sequences of 96,437 SARS-CoV-2 strains were downloaded from the GISAID database. We gratefully acknowledge the authors from the originating laboratories responsible for obtaining these specimens and the submitting laboratories where genetic sequence data were generated and shared via the GISAID initiative^36,37^. Supplementary Data 4 provides a complete list of these strains, authors, and laboratories used in this manuscript.

### SARS-CoV-2 strain analysis

For each of the 96,437 SARS-CoV-2 proteomes, we identified amino acid mutations relative to the original SARS-CoV-2 strain (hCoV-19/Wuhan/WIV04/2019). Incomplete proteomes were not considered. A total of 21,127 unique amino acid mutations were identified across spike glycoprotein, membrane protein, envelope protein, and nucleoprotein. For each mutation, a region of 10 flanking amino acids on either side was considered as the mutated region, for comparison against the same length wild-type region. For every sample, we calculated and compared PIWAS scores for the wild-type and mutant sequences. To assess significance of the observed bias, we generated in silico random mutations to these same proteins and performed the same analysis. We compared the actual and random signals using a Kolmogorov-Smirnov test.

### Construction and screening of a SARS-CoV-2-specific peptide library

An *Escherichia coli* bacterial display library encompassing the entire SARS-CoV-2 proteome was constructed using a surface display vector carrying linear peptides derived from the SARS-CoV-2 proteome (Genbank MN908947.3). Designed oligonucleotides (Twist Bioscience) encoded peptides 12 amino acids in length and tiled with 8 amino acids overlap. SARS-CoV-2 sera and controls were screened with the *SERA serum screening* protocol as described above, with the following modifications. Serum samples (0.5 µL each) were diluted 1:200 in a suspension of PBS and bacteria carrying the surface display library (10^9^ cells per sample with 3 × 10^5^ fold library representation), and incubated. After centrifugation and washing to remove unbound antibody, 5 µL of protein A/G magnetic beads diluted 1:10 in PBS were incubated with the library to pull down the antibody-bound bacteria. Unique molecular identifiers (UMI) were applied during PCR to minimize amplification bias, designed as an 8 base pair semi-random sequence (NNNNNNHH). After preprocessing and read trimming the raw sequencing data, the resulting reads were filtered by utilizing the UMIs to remove PCR duplicates. The filtered UMI data were then aligned to the original reference of linear epitopes derived from SARS-CoV-2 and quantified.

### Antibodies

The secondary antibody used for IgM antibody screening: Biotin-SP (long spacer) AffiniPure F(ab’)2 Fragment Donkey Anti-Human IgM, Fc5u fragment specific (min X Bov, Hrs Sr Prot) from Jackson ImmunoResearch, Code 709-066-0739, Lot: 147595. The antibody was used at 1:100 final dilution. The IgM secondary antibody is commercially available and validated by the manufacturer. Additionally, we have performed internal quality control analysis with healthy control serum samples serving as run standards across biological and technical replicates to validate secondary antibody performance in our assay.

### Reporting summary

Further information on research design is available in the Nature Research Reporting Summary linked to this article.

## Supplementary information

Supplementary Information

Description of Additional Supplementary Files

Supplementary Data 1

Supplementary Data 2

Supplementary Data 3

Supplementary Data 4

Reporting Summary

## Data Availability

The datasets including motif enrichment and PIWAS data generated and analyzed during this study (Figs. 2–6), and Supplemental Data files have been made available at: http://www.serimmune.com/covidData.tgz.

## Code availability

All code was written in R(3.6.3)^65^. The code used to generate all figures (including statistical tests) has been made available at: http://www.serimmune.com/covidData.tgz.
