# Supplementary material for: High-resolution epitope mapping and characterization of SARS-CoV-2 antibodies in large cohorts of subjects with COVID-19
Source: Commun Biol. 2021 Nov 22;4:1317. doi: 10.1038/s42003-021-02835-2 (PMC8608966; doi:10.1038/s42003-021-02835-2)

Supplementary Figure 1: A comparison of PIWAS tiling signal to antigens from SARS-CoV-2 and the SARS-CoV-2-specific peptide library signal to each peptide from the SARS-CoV-2 proteome. Locations where PIWAS values over 2.5 (black dashed line) have been marked with a purple dashed line in both the PIWAS plot, and the SARS-CoV-2-specific library plots.

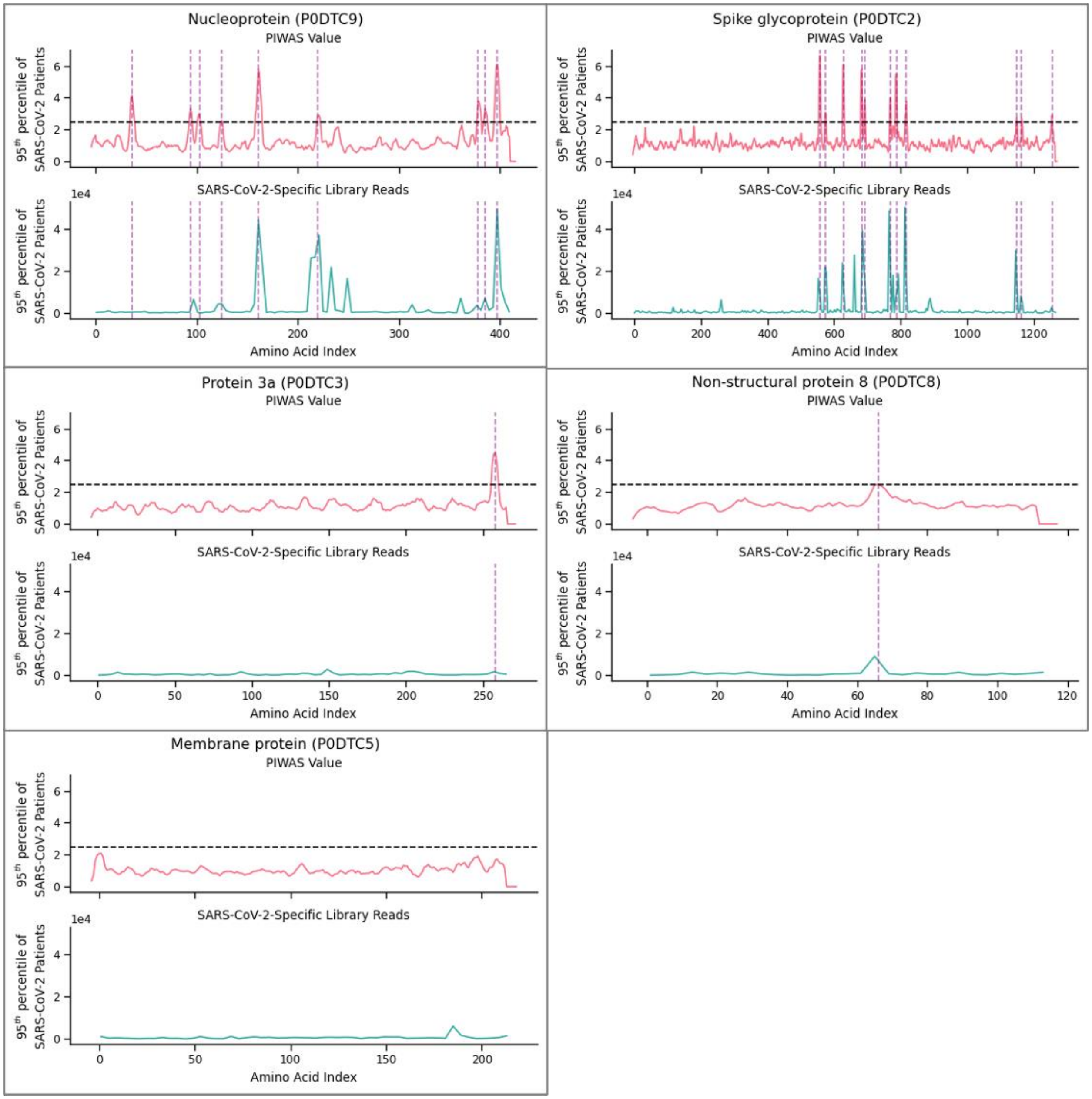

Supplementary Figure 2: Number of COVID-19 samples with outlier PIWAS signal across SARS-CoV-2 proteins.

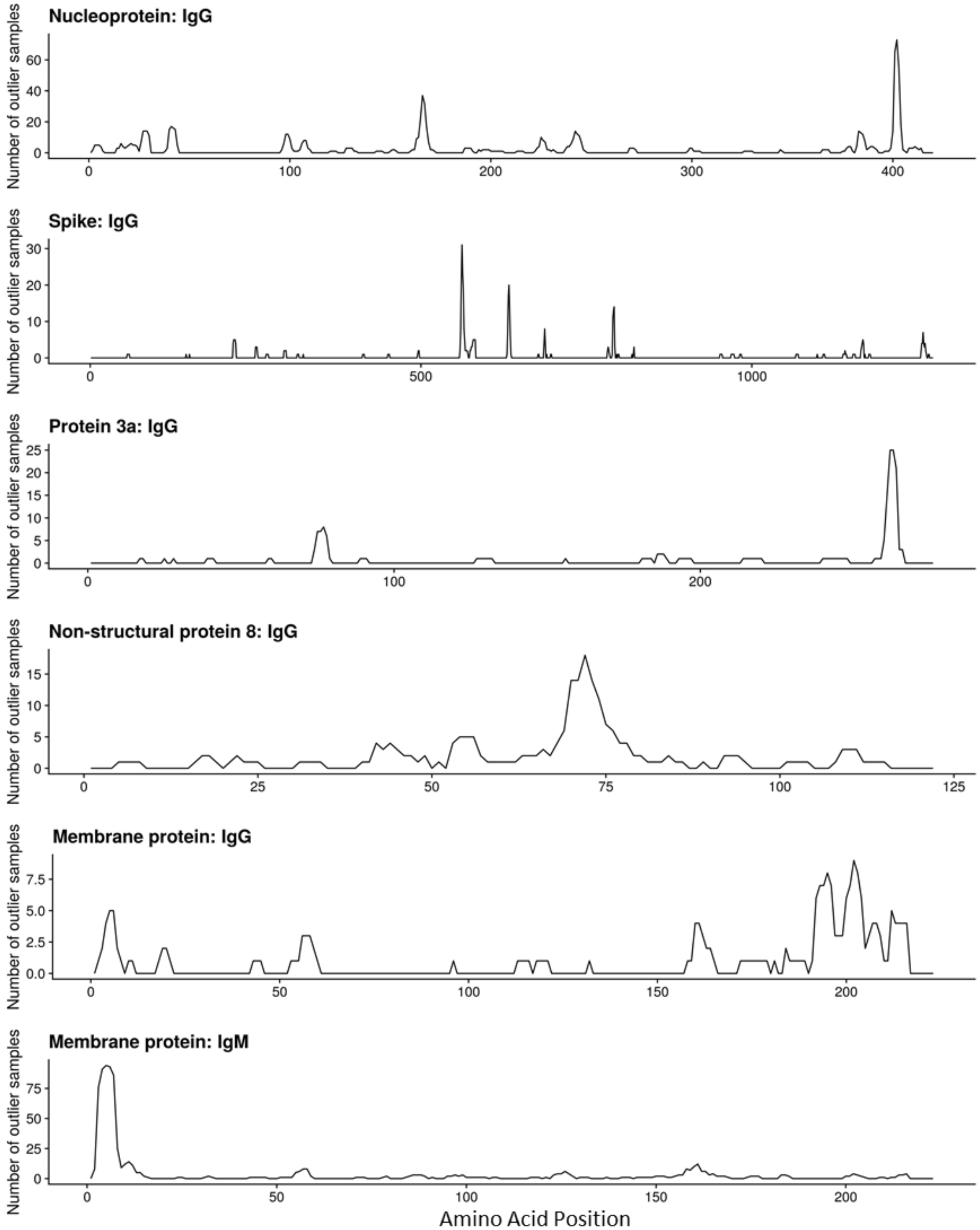

Supplementary Figure 3: Correlation of COVID-19 motif enrichment with neutralization titers.

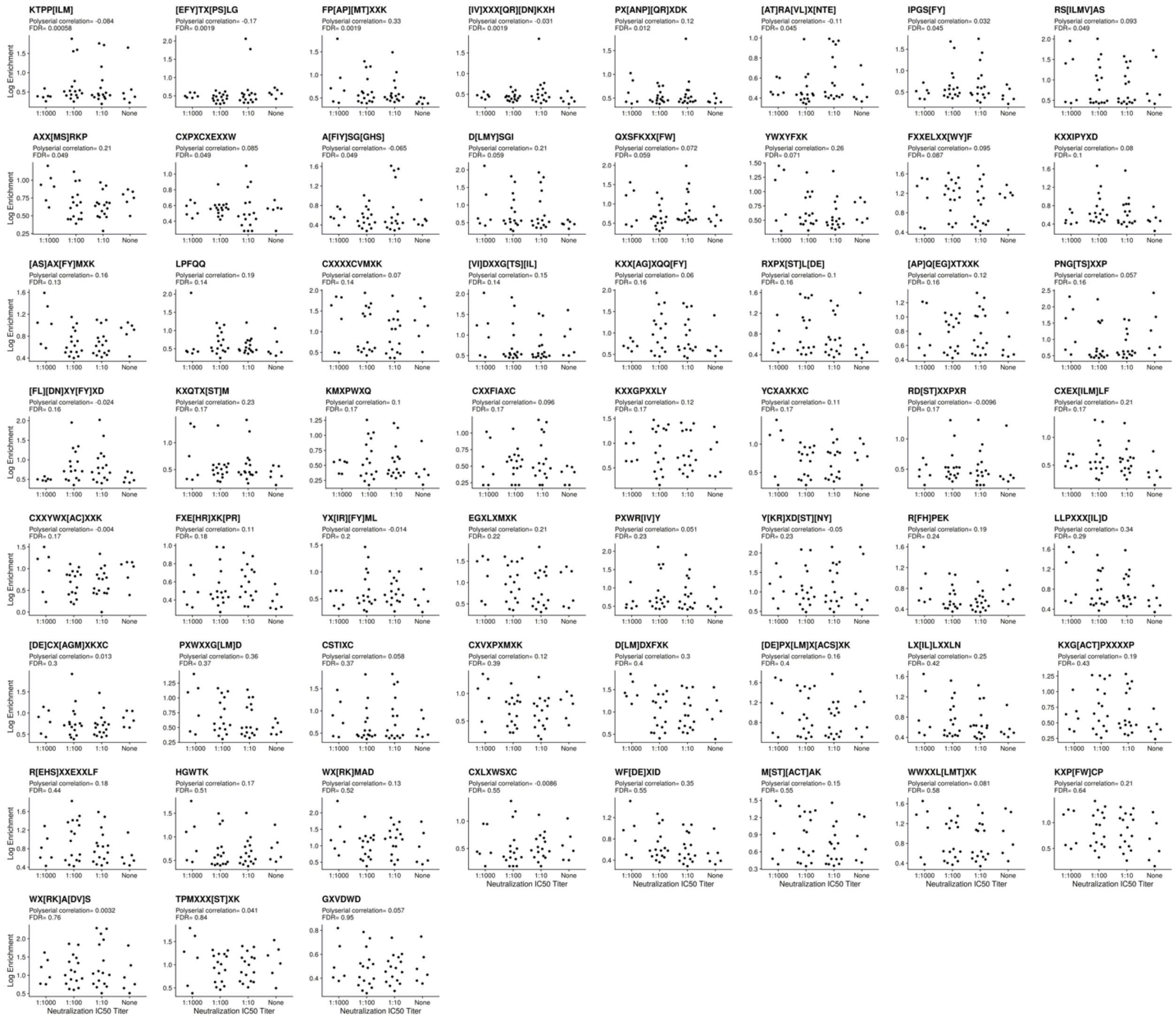

Supplementary Figure 4: Association of COVID-19 motifs with disease severity.

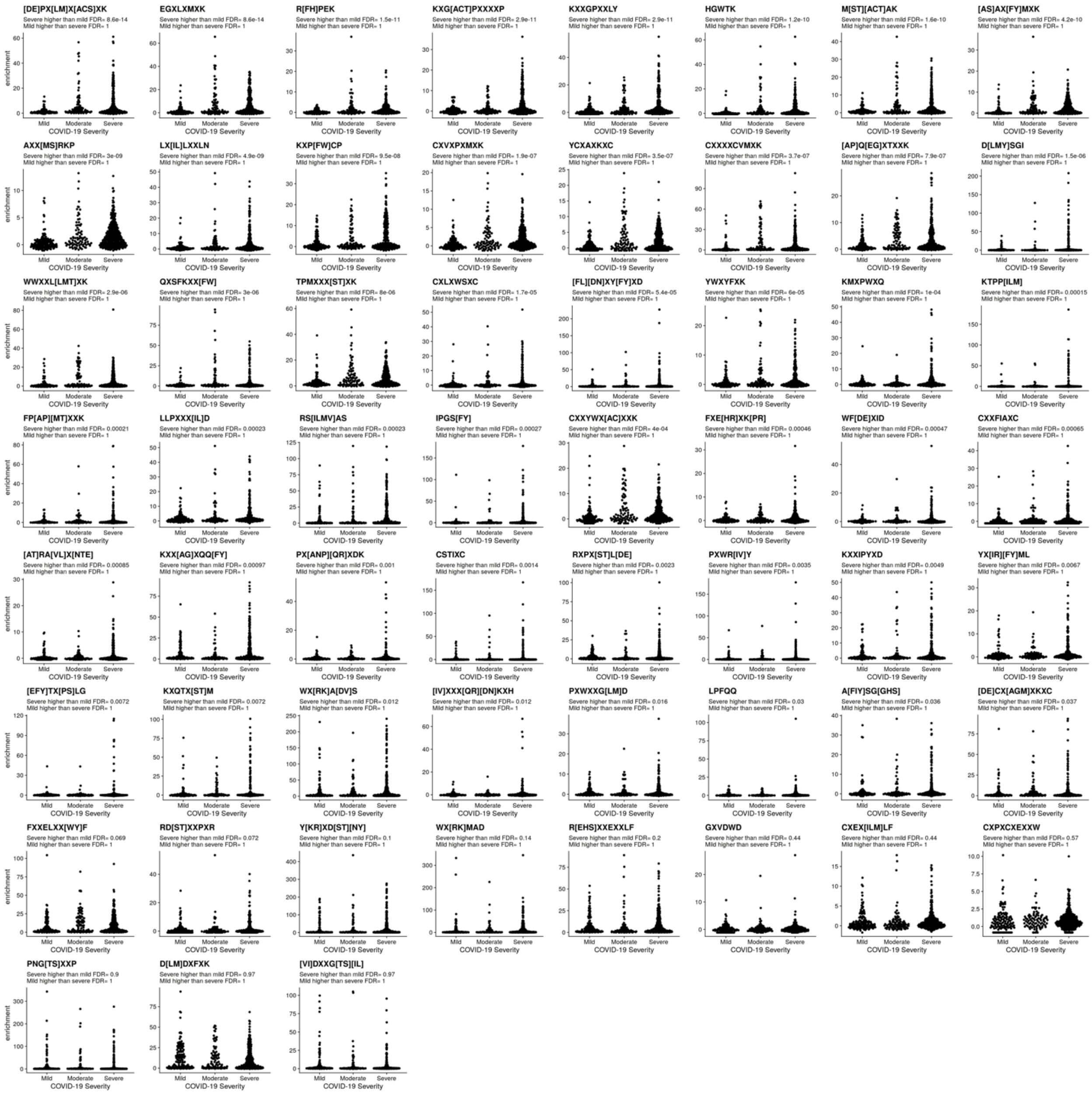

Supplementary Figure 5: Classification of COVID-19 patients by severity using motif-based panel.

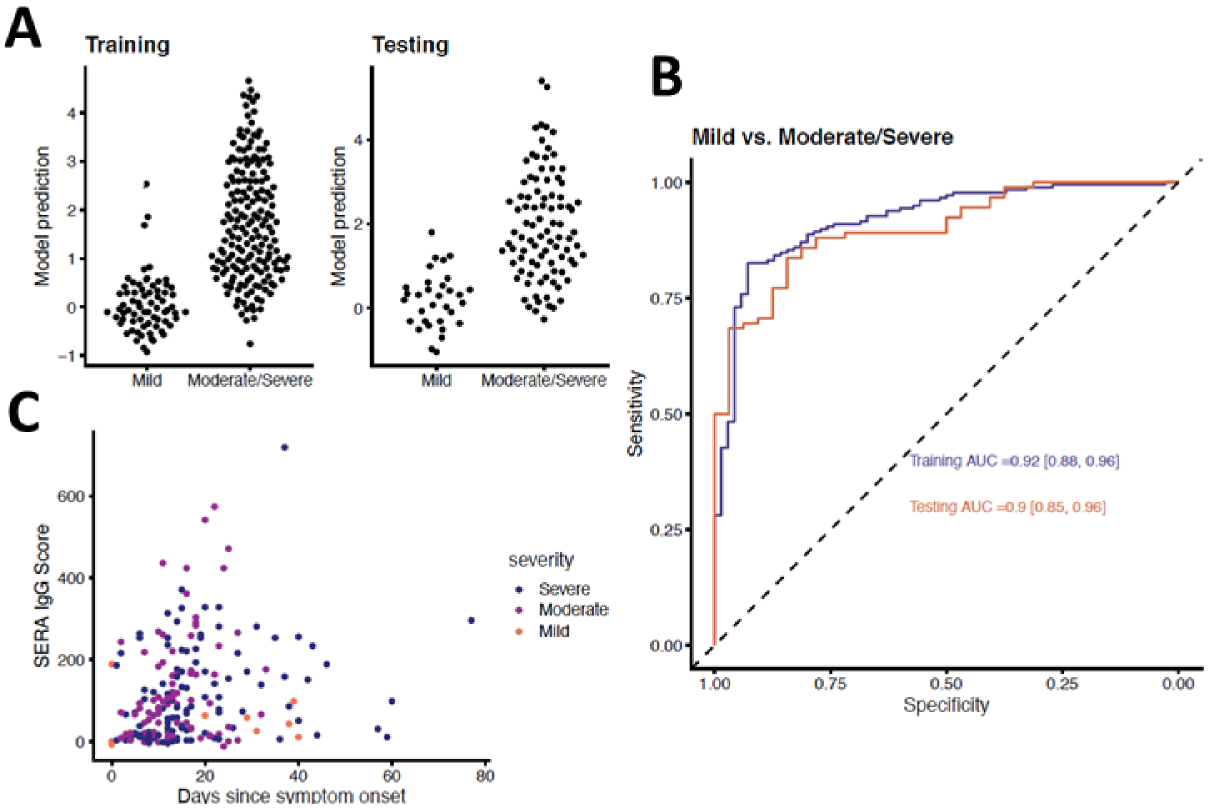

Supplementary Figure 6: Distributions of frequency and samples with significant changes in PIWAS values for observed and random mutations.

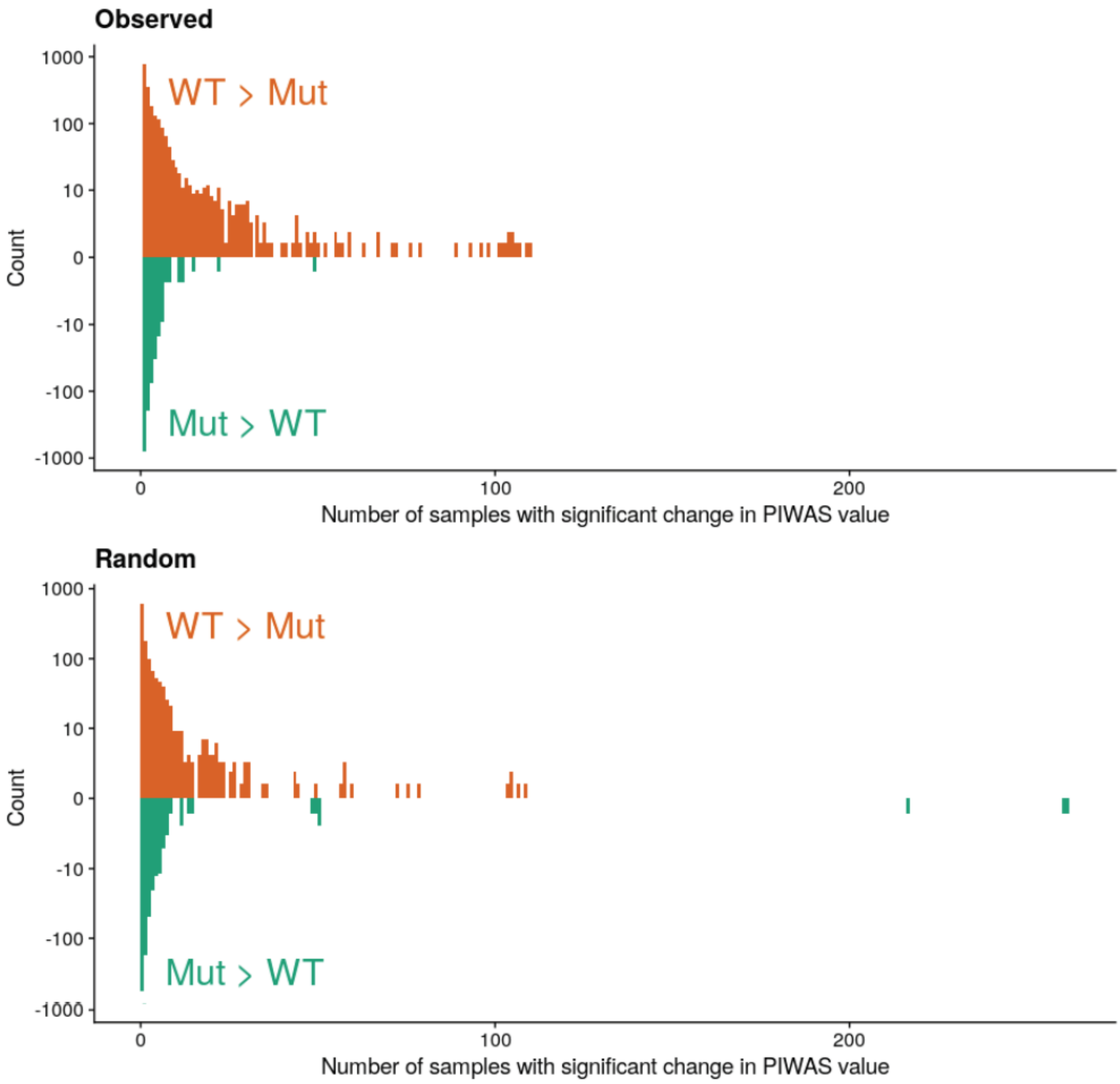

Supplement: Supplementary file 1 — Supplementary Information [file 42003_2021_2835_MOESM1_ESM.pdf]
