# Supplementary material for: High-resolution epitope mapping and characterization of SARS-CoV-2 antibodies in large cohorts of subjects with COVID-19
Source: Commun Biol. 2021 Nov 22;4:1317. doi: 10.1038/s42003-021-02835-2 (PMC8608966; doi:10.1038/s42003-021-02835-2)
Supplement: Supplementary file 2 — Description of Additional Supplementary Files [file 42003_2021_2835_MOESM2_ESM.pdf]

Description of Additional Supplementary Files

File name: Supplementary Data 1
Description: COVID-19 Sample Information.

File name: Supplementary Data 2
Description: Epitopes in the random and focused libraries.

File name: Supplementary Data 3
Description: Sensitivity and specificity of IMUNE epitope motifs in SERA or ELISA positive SARS-CoV-2.

File name: Supplementary Data 4
Description: GISAID strains data.
