# Supplementary material for: High-resolution epitope mapping and characterization of SARS-CoV-2 antibodies in large cohorts of subjects with COVID-19
Source: Commun Biol. 2021 Nov 22;4:1317. doi: 10.1038/s42003-021-02835-2 (PMC8608966; doi:10.1038/s42003-021-02835-2)
Supplement: Supplementary file 7 — Reporting Summary [file 42003_2021_2835_MOESM7_ESM.pdf]

nature portfolio

Corresponding author(s): John C. Shon

Last updated by author(s): Oct 1, 2021

Reporting Summary

Nature Portfolio wishes to improve the reproducibility of the work that we publish. This form provides structure for consistency and transparency in reporting. For further information on Nature Portfolio policies, see our Editorial Policies and the Editorial Policy Checklist.

Statistics

For all statistical analyses, confirm that the following items are present in the figure legend, table legend, main text, or Methods section.

n/a	Confirmed	
☐	☒	The exact sample size (*n*) for each experimental group/condition, given as a discrete number and unit of measurement
☐	☒	A statement on whether measurements were taken from distinct samples or whether the same sample was measured repeatedly
☐	☒	The statistical test(s) used AND whether they are one- or two-sided<br>*Only common tests should be described solely by name; describe more complex techniques in the Methods section.*
☐	☒	A description of all covariates tested
☐	☒	A description of any assumptions or corrections, such as tests of normality and adjustment for multiple comparisons
☐	☒	A full description of the statistical parameters including central tendency (e.g. means) or other basic estimates (e.g. regression coefficient) AND variation (e.g. standard deviation) or associated estimates of uncertainty (e.g. confidence intervals)
☐	☒	For null hypothesis testing, the test statistic (e.g. *F*, *t*, *r*) with confidence intervals, effect sizes, degrees of freedom and *P* value noted<br>*Give P values as exact values whenever suitable.*
☒	☐	For Bayesian analysis, information on the choice of priors and Markov chain Monte Carlo settings
☒	☐	For hierarchical and complex designs, identification of the appropriate level for tests and full reporting of outcomes
☒	☐	Estimates of effect sizes (e.g. Cohen's *d*, Pearson's *r*), indicating how they were calculated

Our web collection on statistics for biologists contains articles on many of the points above.

Software and code

Policy information about availability of computer code

Data collection	Data was collected as previously described in https://www.nature.com/articles/srep30312
Data analysis	All code was written in R(3.6.3). The code used to generate all figures (including statistical tests) has been made available at: https://bitbucket.org/serimmunedatascience/sarscov2-epitopes-manuscript/

For manuscripts utilizing custom algorithms or software that are central to the research but not yet described in published literature, software must be made available to editors and reviewers. We strongly encourage code deposition in a community repository (e.g. GitHub). See the Nature Portfolio guidelines for submitting code & software for further information.

Data

Policy information about availability of data

All manuscripts must include a data availability statement. This statement should provide the following information, where applicable:

- Accession codes, unique identifiers, or web links for publicly available datasets
- A description of any restrictions on data availability
- For clinical datasets or third party data, please ensure that the statement adheres to our policy

The datasets including motif enrichment and PIWAS data generated and analyzed during this study (Figures 2-6) have been made available at: https://www.serimmune.com/covidData.zip

Field-specific reporting

Please select the one below that is the best fit for your research. If you are not sure, read the appropriate sections before making your selection.

[x] Life sciences [ ] Behavioural & social sciences [ ] Ecological, evolutionary & environmental sciences

For a reference copy of the document with all sections, see nature.com/documents/nr-reporting-summary-flat.pdf

Life sciences study design

All studies must disclose on these points even when the disclosure is negative.

Sample size

Sample sizes for COVID-19 cohorts were not calculated based on statistical methods and were based on the number of subjects and samples available from each study/cohort. Sample sizes for pre-pandemic controls were driven by statistical calculations for PIWAS analysis, and a limit of at least 500 control samples has been established. We used 1,500 control samples given the availability of additional pre-pandemic controls.

Data exclusions

No data were excluded from these analyses.

Replication

Extensive internal validation for the SERA assay, including technical and biological variation, has been completed, including a precision study of COVID-19 serum samples with respect to SARS-CoV-2 epitope enrichment. Additionally, every 96-well plate of samples processed for this study contained healthy control run standards to assess and evaluate assay reproducibility and possible batch effects. The COVID-19 diagnostic panels developed in this study were trained on discovery cohorts and validated on independent testing cohorts (Table 1 of manuscript). Other findings were not explicitly replicated.

Randomization

Subject samples were stratified based on COVID-19 diagnosis and disease severity. COVID-19 diagnosis was determined by positive PCR test and/or ELISA for SARS-CoV-2, and controls were restricted to samples collected prior to the SARS-CoV-2 outbreak (pre-pandemic). When available, COVID-19 samples were stratified by disease severity. From the Yale cohort, cases were classified as mild if patients were not hospitalized, moderate if hospitalized, and severe if on high-flow nasal canula, BiPAP or other non-invasive ventilation, intubated or died from COVID-19. Fro the SBCH cohort, cases were classified as asymptomatic, mild/moderate if the subject had symptoms consistent with acutely ill and ICU hospitalized. For the BioIVT cohort, samples were classified as mild or severe characterized by the commercial vendor and clinical associates. For the BCA cohort, cases were identified from assumed healthy blood donors, later revealed to test positive for SARS-CoV-2 via S1 ELISA, and therefore classified as mild disease. Samples were obtained and analyzed from multiple cohorts to reduce any cohort-dependent effects.

Blinding

At the time of sample acquisition, samples were designated with generic sample IDs and scientists were unaware of sample cohort status during sample processing

Reporting for specific materials, systems and methods

We require information from authors about some types of materials, experimental systems and methods used in many studies. Here, indicate whether each material, system or method listed is relevant to your study. If you are not sure if a list item applies to your research, read the appropriate section before selecting a response.

Materials & experimental systems

n/a	Involved in the study	
[ ]	[x]	Antibodies
[x]	[ ]	Eukaryotic cell lines
[x]	[ ]	Palaeontology and archaeology
[x]	[ ]	Animals and other organisms
[ ]	[x]	Human research participants
[x]	[ ]	Clinical data
[x]	[ ]	Dual use research of concern

Methods

n/a	Involved in the study	
[x]	[ ]	ChIP-seq
[x]	[ ]	Flow cytometry
[x]	[ ]	MRI-based neuroimaging

Antibodies

Antibodies used

The secondary antibody used for IgM antibody screening: Biotin-SP (long spacer) AffiniPure F(ab')2 Fragment Donkey Anti-Human IgM, Fc5u fragment specific (min X Bov, Hrs Sr Prot) from Jackson ImmunoResearch, Code 709-066-0739, Lot: 147595. The antibody was used at 1:100 final dilution.

Validation

The IgM secondary antibody is commercially available and validated by the manufacturer. Additionally, we have performed internal quality control analysis with healthy control serum samples serving as run standards across biological and technical replicates to validate secondary antibody performance in our assay.

Human research participants

Policy information about studies involving human research participants

Population characteristics	Sex, n, average age, range<br>Male, 210, 57, 5 to 94<br>Female, 200, 61, 18 to 95<br>410 total
Recruitment	Yale IMPACT cohort<br>Patients admitted to the Yale New Haven Hospital (YNHH) were recruited to the Yale IMPACT study (Implementing Medical and Public Health Action against Coronavirus CT) after testing positive for SARS-CoV2 by qRT-PCR or after a suspected COVID-19 diagnosis followed by a positive SARS-CoV-2 serology test. Patients were identified through screening of EMR records for potential enrollment with no self-selection. Informed consent was obtained by trained staff and sample collection commenced immediately upon study enrollment. Health care workers were recruited as part of a longitudinal monitoring study. Subjects with a positive SARS-CoV-2 qRT-PCR test of positive serology were included in this study.<br>SBCH cohort<br>Biobanked sera or plasma from individuals that previously tested positive for COVID_19 by a nucleic acid test (NAT) or exhibiting symptoms consistent with COVID_19 were provided by the Santa Barbara Cottage Hospital Biobank. Diagnosis of COVID_19 in subjects with an unknown or negative SARS-CoV-2 NAT test was confirmed by serology.
Ethics oversight	The Yale IMPACT biorepository study was approved by Yale Human Research Protection Program Institutional Review Boards (protocol ID 2000027690). Santa Barbara Cottage Health IRB approved the sample collection for the SBCH cohort with human subject research exemption. All other samples included in this study were remnant de-identified samples.

Note that full information on the approval of the study protocol must also be provided in the manuscript.
